# A data-driven measure of REM sleep propensity for human and rodent sleep

**DOI:** 10.3389/fnins.2026.1844209

**Published:** 2026-06-09

**Authors:** Naghmeh Akhavan, Alexander G. Ginsberg, Madelyn E. C. Cruz, Yunxi Yan, Shelby R. Stowe, Dinesh Pal, Franz Weber, Cecilia G. Diniz Behn, Victoria Booth

**Affiliations:** 1 Department of Mathematics, University of Michigan, Ann Arbor, MI, United States; 2 Department of Mathematics, University of Utah, Salt Lake City, UT, United States; 3 Department of Applied Mathematics and Statistics, Colorado School of Mines, Golden, CO, United States; 4 Department of Anesthesiology, University of Michigan, Ann Arbor, MI, United States; 5 Department of Neuroscience, University of Pennsylvania, Philadelphia, PA, United States; 6 Department of Pediatrics, University of Colorado Anschutz Medical Campus, Aurora, CO, United States

**Keywords:** hourglass process, NREM-REM sleep cycles, REM sleep pressure, sequential REM sleep episodes, sleep cycle, ultradian rhythms

## Abstract

**Introduction:**

Mammalian sleep is characterized by alternations between episodes of rapid-eye-movement sleep (REMS) and non-REM sleep (NREMS). The phenomenon of REMS pressure, namely a drive for REMS that builds up between REMS episodes, is thought to govern the timing of these ultradian NREMS-REMS cycles. Prior analyses of NREMS-REMS cycles in mice suggested that time in NREMS is a primary contributor to REMS pressure. We previously introduced a REMS propensity measure defined as the probability to enter REMS before the accumulation of an additional amount of NREMS. Analyzing mouse sleep data, we showed that REMS propensity at REMS onset was positively correlated with REMS bout duration and with the probability of the occurrence of a REMS bout followed by a short inter-REMS interval, called a sequential REMS cycle.

**Methods:**

Here, we extend the analysis of NREMS-REMS cycling to human and rat sleep behavior. We compare REMS propensity measures computed from sleep data recorded in humans, mice, and rats. As REMS in humans is influenced by the circadian rhythm, we also analyze circadian modulation of the expression of NREMS-REMS cycles across the human sleep episode.

**Results:**

We find that, as in mice, human and rat sleep contain both short sequential NREMS-REMS cycles and longer single NREMS-REMS cycles, with differences in the timescales of cycle durations. Although rodents exhibit polyphasic sleep in contrast with the consolidated sleep of humans, the calculated REMS propensity measures in all three species show similar profiles as functions of time spent in NREMS. Importantly, positive correlations of REMS propensity at REMS onset with REMS bout duration were present in both human and rat data as previously found in mouse data, suggesting that time spent in NREMS also influences REMS duration in these species. In the human data, we identified nuanced changes in the occurrence of single and sequential NREMS-REMS cycles suggesting that increased percent time spent in REMS as the sleep episode progresses is not solely due to increased REMS bout duration.

**Conclusion:**

Results suggest that similarities in the regulation of NREMS-REMS alternation exist, despite temporal differences, in nocturnal polyphasic rodent sleep and diurnal monophasic human sleep.

## 1 Introduction

In mammals, sleep alternates between episodes of rapid-eye-movement sleep (REMS) and non-REM sleep (NREMS), often referred to as ultradian cycling. Although much progress has been made in identifying the hypothalamic and brainstem areas and circuits that are involved in promoting or suppressing REMS (Luppi et al., 2024), the mechanisms governing the timing of ultradian alternation between NREMS and REMS remain poorly understood (Le Bon, 2021).

One feature of NREMS-REMS alternation is a bimodal distribution of inter-REMS intervals, defined as the durations of intervals between successive REMS bouts (Zamboni et al., 1999; Park et al., 2021; Ursin, 1970; Kripke et al., 1968; Gregory and Cabeza, 2002; Amici et al., 1994). In rats and mice, the bimodal distribution of inter-REMS intervals has led to the identification of two distinct types of NREMS-REMS cycles: *single REMS cycles*, defined as REMS bouts that are preceded and followed by longer inter-REMS intervals, and *sequential REMS cycles*, defined as REMS bouts that are separated by shorter inter-REMS intervals and can occur consecutively in sequences (Zamboni et al., 1999; Park et al., 2021). In human sleep, a similar bimodal distribution of inter-REMS intervals has been observed (Merica and Gaillard, 1991; Kobayashi et al., 1985; Esposito et al., 2003, 2004), however definitions for sequential cycles have differed from those proposed for rodent sleep (Merica and Gaillard, 1991; Esposito et al., 2004). Moreover, in human sleep, it has been a common sleep scoring practice to combine series of consecutive REMS bouts that occur in quick succession (typically separated by less than 15 min of NREMS, wake, or movement) into one, consolidated REMS bout (Merica and Gaillard, 1991). This scoring practice obscures fragmentation within “consolidated” REMS episodes that is typical in healthy human sleep. However, it remains unclear if these periods of fragmented REMS are comparable to the sequential REMS cycles observed in rodent sleep.

An active hypothesis for a mechanism governing the timing of NREMS-REMS alternation posits that a short-term homeostatic drive or REMS pressure contributes to the initiation of REMS (Benington and Heller, 1994b,a; Vivaldi et al., 1994; Zamboni et al., 1999). Specifically, a drive for REMS builds up between REMS bouts and discharges during REMS, often referred to as “hourglass-like” dynamics (Zamboni et al., 1999; Park et al., 2021; Craig, 2021). This theory is supported by wide-spread evidence that longer REMS bouts are followed by longer intervals before the next REMS bout occurs (Barbato and Wehr, 1998; Vivaldi et al., 1994; Benington et al., 1994; Vivaldi et al., 2005; Park et al., 2021; Cajochen et al., 2024), presumably because more of the drive for REMS is discharged during a longer REMS episode. However, the biological substrate mediating this growth and decay of REMS pressure is‘unknown.

Various measures that may reflect REMS pressure have been proposed, including the percent time spent in REMS, average latency to the first REMS episode and the occurrence rate of longer REMS episodes (Czeisler et al., 1980b; Ocampo-Garcés et al., 2013; Nielsen et al., 2010). Other studies have measured REMS propensity based on the overall rate of transitions from NREMS to REMS (Bassi Acuña et al., 2009; Werth et al., 2002) or on the temporal architecture of NREMS-REMS transitions such as the number of brief (< 30 s) REMS episodes occurring between sustained (>30 s) REMS episodes (Benington et al., 1994). In an analysis of spontaneous sleep in mice, Park et al. (2021) proposed a data-driven, probabilistic measure of propensity for REMS, namely the cumulative distribution function (CDF) of the amount of NREMS between REMS bouts. They found that the CDF of the amount of NREMS between REMS bouts was predictive for the duration of REMS episodes (Park et al., 2021). However, in a quantitative sense, REMS pressure should dictate the propensity or probability of entering REMS at a specific time during a sleep episode. Therefore, our group recently proposed an alternative REMS propensity measure, *P*(*t*, Δ), defined as the probability that, after the accumulation of *t* s of NREMS since the last REMS bout, a transition to REMS will occur within the next Δ s spent in NREMS (Ginsberg et al., 2024). Thus, *P*(*t*, Δ) was the first predictive measure of REMS pressure that is readily interpretable as the probability of entering REMS at a certain time during a sleep episode. Computing our REMS propensity measure from the same spontaneous sleep data in mice as was used in the Park et al. study (Park et al., 2021), we showed that, as the amount of time spent in NREMS increases, this REMS propensity measure increases until it reaches a peak value. After this point, REMS propensity eventually decays to zero as the time spent in NREMS continues to accumulate. We found that during the light phase, this REMS propensity measured at REMS onset was positively correlated with the duration of the REMS bout (Ginsberg et al., 2024). Further, higher propensities following single REMS cycles (Zamboni et al., 1999; Park et al., 2021) were correlated with a higher probability of being followed by a sequential REMS cycle (Zamboni et al., 1999; Park et al., 2021; Ursin, 1970; Merica and Gaillard, 1991; Kripke et al., 1968; Gregory and Cabeza, 2002; Amici et al., 1994). However, after the propensity reaches its peak value, its correlation with the features of the subsequent REMS bout was lost, suggesting that the amount of time in NREMS drives transitions into REMS only for a limited range of NREMS accumulation.

In this paper, we extend our analysis of REMS propensity to (diurnal) human and (nocturnal) rat sleep data and present it in the context of the original mouse data from the Weber lab (Park et al., 2021). We used a comparable set of rat sleep data recorded in the Pal lab (Silverstein et al., 2024). For the human data, we utilized three publicly available human sleep datasets (Lopez-Larraz et al., 2024; Goldberger et al., 2000; Zhang et al., 2018) comprised of five study cohorts. These datasets were collected in participants representing a range of demographic groups, over different time periods, under different inclusion/exclusion criteria, and with different experimental and scoring protocols (for details, see Methods and Lopez-Larraz et al., 2024; Goldberger et al., 2000; Zhang et al., 2018; Kemp et al., 2000; Stephansen et al., 2018).

For our analysis, we first compare characteristics of NREMS-REMS alternation across human, mouse and rat species. Then we show that the bimodal distribution of time spent in NREMS between REMS bouts in human and rat data can be fit with mixture models (MMs). The rat data is fit well with a Gaussian mixture model, similar to the mouse data, while the human data is fit with a three part mixture model.

Using the MM fits, we compute the REMS propensity functions for each species. Despite differences in the underlying mixture models, the resulting propensity functions show a similar qualitative profile across species: they increase with time spent in NREMS up to a peak value and then decrease with further NREMS accumulation. We show that in all three species the duration of a REMS bout is positively correlated with the REMS propensity value at REMS onset, before the propensity reaches its peak value.

The circadian timing of REMS varies between nocturnal and diurnal animals with REMS most common during the inactive period, i.e., during the light (dark) phase for nocturnal (diurnal) animals. Additionally, circadian modulation of REMS occurrence has been observed across the nighttime sleep episode in humans (Czeisler et al., 1980a; Dijk and Czeisler, 1995; Dijk et al., 2010) and to a lesser extent across the light phase in rodents (Borbély, 1980; Benington and Heller, 1994b; Yasenkov and Deboer, 2012). To establish circadian features of REMS expression in the human data, we analyzed how the occurrence of sequential and single REMS cycles evolves across the sleep episode. We find that sequential cycles occur more often at the very beginning and at the very end of the sleep episode, revealing nuanced changes in REMS micro-architecture that contribute to an increase in percent time spent in REMS as the sleep episode progresses.

Overall, our results highlight similarities in NREMS-REMS alternation across three mammalian species, human, rat, and mouse, regardless of the temporal differences between nocturnal polyphasic rodent sleep and diurnal monophasic human sleep. Furthermore, our work shows how these similarities may be masked when sequential REMS cycles are combined following standard practices for scoring human sleep.

## 2 Results

**Figure 1:**
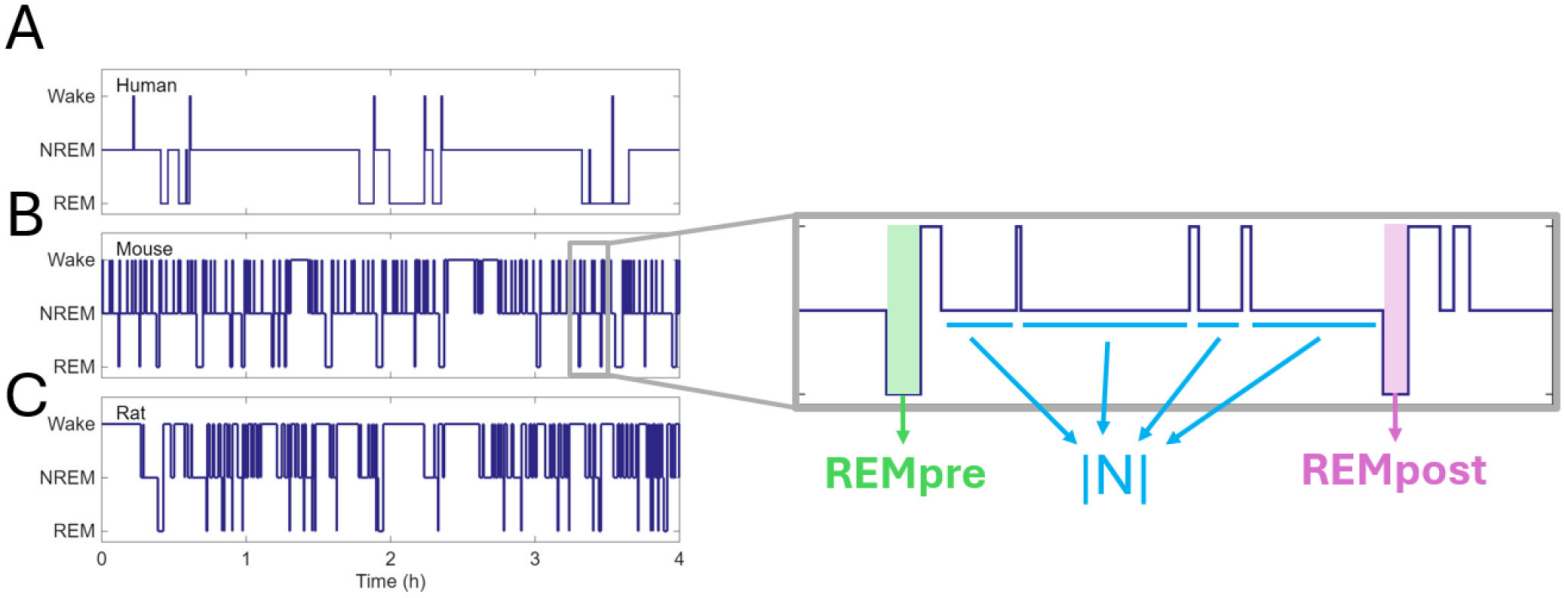
Example hypnograms of sleep behavior over 4 h in human **(A)**, mouse [**(B)**, light period] and rat [**(C)**, light period]. The inset highlights a representative REMS cycle that is initiated at the onset of a REMS bout, REMpre (green), and terminates at the onset of the next REMS bout, REMpost (magenta). The total time spent in NREMS during the inter-REMS interval is defined as |*N*| (blue). Note that the full inter-REMS interval contains both NREMS and wake bouts shorter than 2 min.

To investigate the conservation of a probabilistic structure of NREMS-REMS cycling across mammalian species, we analyzed sleep-scored data from human, mouse and rat sleep recordings using a unified computational framework. For each species, we segmented sleep into successive REMS cycles, each comprised of an initial REMS bout, REMpre, followed by an inter-REMS interval containing NREMS and wake states (human data may also contain epochs scored as movement) (Figure 1). To distinguish overall cycle timing from the amount of NREMS that we posit is specifically relevant to REMS pressure, we consider two related durations within each REMS cycle. We define the duration of the inter-REMS interval, denoted by |IREM|, as the total time from the end of one REMS bout to the onset of the next REMS bout. This interval includes all intervening states, including NREMS and any brief wake or movement periods that do not meet the long-wake exclusion criterion (see below). Within this interval, we define |*N*| as the cumulative time spent in NREMS only. Thus |*N*| ≤ |IREM|, with equality only when no wake or movement occurs in the interval. We consider both quantities because they address different biological questions: |IREM| characterizes the overall spacing of REMS bouts and the temporal structure of ultradian cycling, whereas |*N*| isolates the NREMS accumulation hypothesized to contribute to REMS pressure. Since our propensity measure is intended to quantify the probability of entering REMS as a function of prior NREMS accumulation, |*N*| is the quantity used in our analysis.

Before performing any statistical or model-based analysis, all datasets were preprocessed using a long-wake (LW) filtering criterion. Because extended spontaneous wake episodes can disrupt the intrinsic timing of NREMS-REMS alternation, we excluded any REMS cycle whose inter-REMS interval contained a contiguous wake segment of at least 2 min. This threshold was applied identically to human data and rodent data (in both light and dark conditions), ensuring consistent cross-species comparisons. The choice of the 2-min cutoff was based on a systematic evaluation of alternative thresholds (2, 5, 7, 10 min) which demonstrated that the LW threshold set to 2 min resulted in the most stable and well-structured distribution of NREMS durations for each species; full justification and sensitivity analyses are provided in Supplementary Section S2. In addition, due to the heterogeneity of participants, protocols, and sleep scoring in the human data, we applied a subject-level outlier filter based on the number of valid REMS cycles exhibited per subject after LW filtering for the human data only. Sleep records with cycle counts outside the interquartile-range criterion (< *Q*_1_−1.5IQR or >*Q*_3_+1.5IQR) were excluded to reduce disproportionate influence from outliers (see Methods section for details).

### 2.1 Basic statistics of NREMS–REMS alternation across species

We first summarize metrics of NREMS–REMS cycling for each species. Considering metrics averaged across individuals (Table 1), the average number of REMS cycles per hour varied across species and light-dark condition, with humans, mice, and rats all exhibiting on the order of 2–6 cycles per hour when averaged per recording. Humans showed 5.04 ± 5.44 REMS episodes per hour, reflecting substantial inter-individual variability. The similar values of the mean and standard deviation reflect a high variability in the average REMS episode rate when fragmented REMS bouts are not consolidated. These cross-species differences in variability should be interpreted with caution. The human dataset included substantially more analyzed recordings than the rodent datasets and was assembled from multiple public cohorts with differing acquisition and scoring protocols. Accordingly, the larger standard deviations observed in the human data likely reflect both genuine heterogeneity in human REMS organization and broader between-recording variability arising from the larger and more heterogeneous dataset, rather than a simple species difference alone. Mice displayed clear light–dark modulation, with higher cycling rates during the light phase (5.04 ± 1.74 cycles/hour) and slower cycling during the dark phase (2.24 ± 0.60 cycles/hour). Rats exhibited comparable cycling rates in both phases, averaging 5.77 ± 1.71 cycles per hour in the light phase and 5.67 ± 1.62 cycles per hour in the dark phase. Mean REMS cycle duration ranged from approximately 10–30 min in rodents to roughly 25–30 min in humans. For rodents, REMS occupied smaller fractions of the cycle duration with NREMS and wake approximately splitting the remainder of the cycle length in the light period. Since wake episodes within a cycle are limited by the 2 min LW threshold, this reflects many brief wake episodes interrupting sleep in rodents. In humans, REMS occupied a larger fraction of the cycle and the inter-REM interval consisted primarily of NREMS.

**Table 1:** REMS cycle summary statistics (in minutes) averaged per recording for each species.

Means of individual recording means						
Species	Number of recordings	REMS episodes per hour	|REMpre|	|*N*|	|IREM|	REMS cycle duration
Human	515	5.04 ± 5.44	10.41 ± 7.68	16.30 ± 20.48	16.80 ± 20.55	27.21 ± 25.85
Mouse (Light)	179	5.04 ± 1.74	1.02 ± 0.23	7.83 ± 2.49	12.43 ± 4.87	13.46 ± 5.03
Mouse (Dark)	54	2.24 ± 0.60	1.10 ± 0.19	10.39 ± 2.92	27.67 ± 7.77	28.77 ± 7.85
Rat (Light)	44	5.77 ± 1.71	1.03 ± 0.37	4.44 ± 1.25	10.51 ± 4.05	11.55 ± 4.17
Rat (Dark)	37	5.67 ± 1.62	1.05 ± 0.36	4.43 ± 1.32	10.66 ± 4.16	11.71 ± 4.25

Values are mean ± standard deviation (SD). “REMS episodes per hour” is computed as (cycle count)/(analyzed time). |REMpre| = duration of the REMS bout initiating the cycle. *N*| = cumulative time in NREMS during the inter-REMS interval (excluding wake and movement; sequential cycles may have |*N*| = 0). |IREM| = total time from the end of the REMS episode initiating the cycle to the beginning of the next REMS episode, including NREMS, wake and all other epochs such as movement. REMS cycle duration = |REMpre| + |IREM|. All datasets were filtered with a long-wake episode threshold of 2 min within the inter-REMS interval.

To assess REMS cycle features at the population level, we also examined statistics when all cycles for a species were pooled together (Table 2). Similar cross-species scaling reappears: per cycle, humans displayed the longest inter-REMS intervals (24.23 ± 35.42 min), compared with 5.88 ± 4.08 min in mice (light phase) and 3.30 ± 3.58 min in rats (light phase). As also apparent in the individual averages, inter-REMS interval duration (|IREM|) and the NREMS-only component (|*N*|) of the inter-REMS interval were especially variable in humans where the SD may exceed the mean, indicating substantial heterogeneity on REM cycle durations. Rodents showed shorter timescales but comparable relative variability in |IREM| and |*N*|: coefficients of variation were approximately 0.7 in mice and 0.9–1.1 in rats, indicating that inter-REMS durations remain heterogeneous even within shorter ultradian cycles.

**Table 2:** REMS cycle summary statistics (in minutes) averaged per cycle for each species.

Pooled REMS cycles for each species					
Species	Total REMS cycles	|REMpre|	|*N*|	|IREM|	REMS cycle duration
Human	2,936/4,426	8.89 ± 9.41	22.68 ± 33.20	24.23 ± 35.42	33.12 ± 39.89
Mouse (Light)	4,005/5,300	0.84 ± 0.74	5.16 ± 3.72	5.88 ± 4.08	6.72 ± 4.57
Mouse (Dark)	739/1,307	0.91 ± 0.68	5.94 ± 3.76	6.82 ± 4.10	7.72 ± 4.53
Rat (Light)	2,089/2,766	0.88 ± 1.03	2.82 ± 3.25	3.30 ± 3.58	4.17 ± 4.07
Rat (Dark)	1,745/2,312	0.89 ± 1.04	2.87 ± 3.27	3.36 ± 3.60	4.24 ± 4.09

For each species and light condition, means and SDs are computed for REMS cycles in all recordings combined together. Total REMS cycles reports (number of REMS cycles after the ≥2-min LW filtering threshold was applied)/(total number of REMS cycles).

The differences between Tables 1, 2 arise from the different weighting schemes used in the two summaries. Table 1 averages recording-level means and therefore gives equal weight to each recording, whereas Table 2 averages across all pooled REMS cycles and therefore gives equal weight to each cycle. As a result, recordings containing larger numbers of cycles contribute more strongly to the pooled averages in Table 2. In rodents, this leads to substantially shorter pooled |IREM| values, indicating that recordings with more frequent REMS cycling tend to have shorter inter-REMS intervals and thus dominate the cycle-level averages. In humans, the opposite pattern is observed, with pooled |IREM| slightly exceeding the mean of recording-level means, suggesting a different relationship between number of cycles and inter-REMS timing across recordings. These differences highlight substantial heterogeneity across recordings and show that recording-level and cycle-level summaries capture distinct aspects of REMS organization.

### 2.2 Relationship between preceding REMS duration and subsequent inter-REMS interval

**Figure 2:**
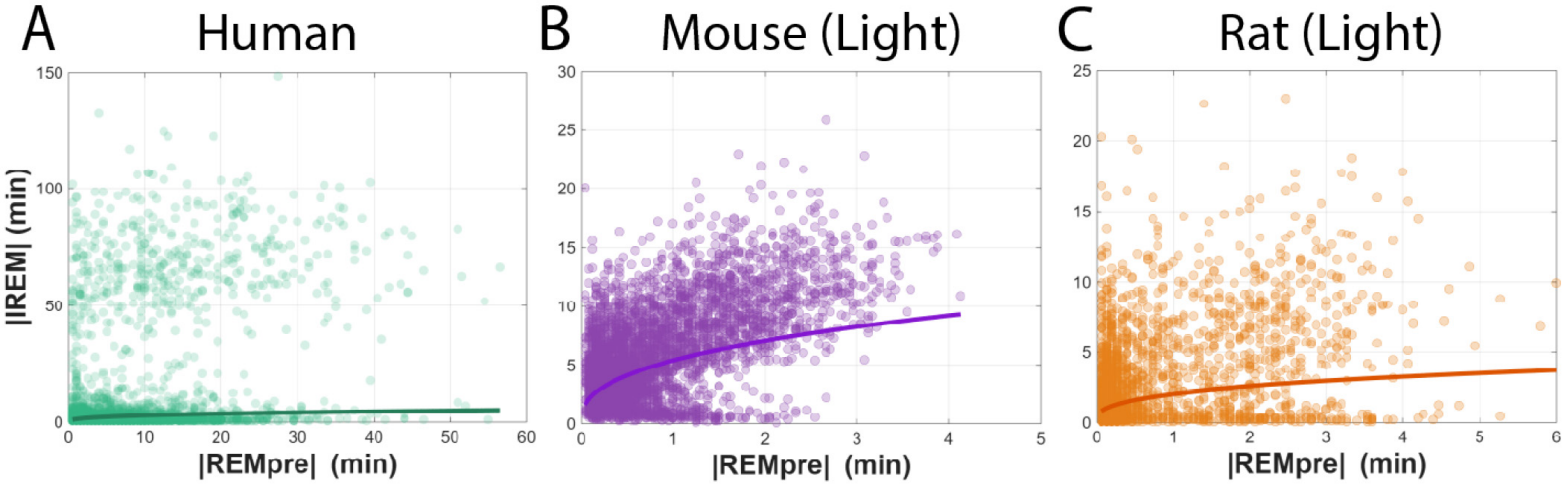
Inter-REMS interval duration increases with the duration of the preceding REMS bout across species. Positive associations are observed between REMS bout duration |REMpre| and the subsequent inter-REMS interval duration |IREM| in all species: **(A)** human (green), **(B)** mouse (light phase, purple), and **(C)** rat (light phase, orange). Each point represents one REMS cycle. Solid lines show log-log linear mixed-effects models fit to the data. The estimated fixed-effect association coefficient for log(|REMpre|) was positive and significant in all three data sets: human (β = 0.333, *p* = 2.92 × 10^−35^), mouse light (β = 0.381, *p* = 9.91 × 10^−171^), and rat light (β = 0.334, *p* = 6.93 × 10^−42^). Mouse and rat dark-phase results are shown in Supplementary Figure S1, where the same positive association was also observed. Details of statistical analyses are provided in Supplementary Section S5.

We next examined whether the duration of a REMS bout influences the length of the subsequent inter-REMS interval, as previously reported (Le Bon, 2021; Vivaldi et al., 2005; Benington and Heller, 1994b) and as expected under a REMS-pressure process with hourglass-type dynamics. Figure 2 compares |REMpre| and the subsequent inter-REMS duration |IREM| across all REMS cycles in each species (see Supplementary Figure S1 for dark-phase rodent data). In these scatter plots, each point represents one REMS cycle, with the x-axis denoting the duration of the REMS bout initiating the cycle (|REMpre|) and the y-axis denoting the duration of the following inter-REMS interval (|IREM|).

Because each subject or animal contributed multiple REMS cycles, we analyzed this relationship using log-log linear mixed-effects models that account for repeated measurements by allowing each subject/animal to have its own baseline level of |IREM|; for the human dataset, data source cohort was additionally included as a fixed effect. Under this framework, we found positive associations between |REMpre| and |IREM| that were significant in all three datasets (Figure 2). The estimated fixed-effect slopes for log(|REMpre|) were positive in humans (β = 0.333), mice in the light phase (β = 0.381), and rats in the light phase (β = 0.334), with all confidence intervals excluding zero. Thus, even after accounting for repeated REMS cycles within subject/animal, longer REMS bouts remained associated with longer subsequent inter-REMS intervals across species. Solid lines in Figure 2 show the population-level fitted trends from these mixed-effects models. Because the analysis was performed on the log-log scale, these positive slopes indicate a sublinear relationship, meaning that |IREM| increases systematically with |REMpre|, but less than proportionally. This association was further evaluated using a Gamma generalized linear mixed-effects (Gamma-GLME) model with log link, and a subject-/animal-level aggregated Spearman correlation analysis. The positive association remained significant in these tests. Together, these results confirm that the duration of a preceding REMS episode predicts the length of the subsequent inter-REMS interval across species, supporting the hypothesis that REMS is regulated by a homeostatic process that resets during each bout (see Supplementary Section S5 for full details of these statistical analyses).

### 2.3 Modeling inter-REMS interval distributions

**Figure 3:**
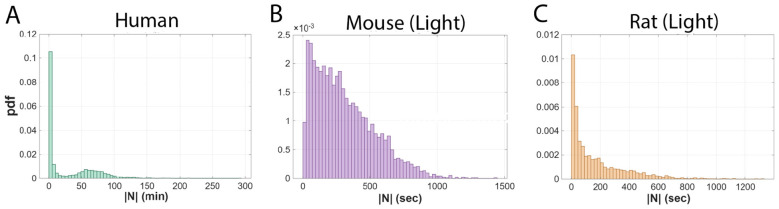
Empirical inter-REMS |*N*| distributions across species suggest multiple characteristic timescales. Histograms show the empirical distribution of |*N*|, where |*N*| is the cumulative duration of NREMS in the inter–REMS interval, for **(A)** human, **(B)** mouse (light phase), and **(C)** rat (light phase) data (see Supplementary Figure S2 for dark phases of rodent data). Across species, |*N*| spans a wide range and exhibits structured, non-Gaussian profiles.

**Figure 4:**
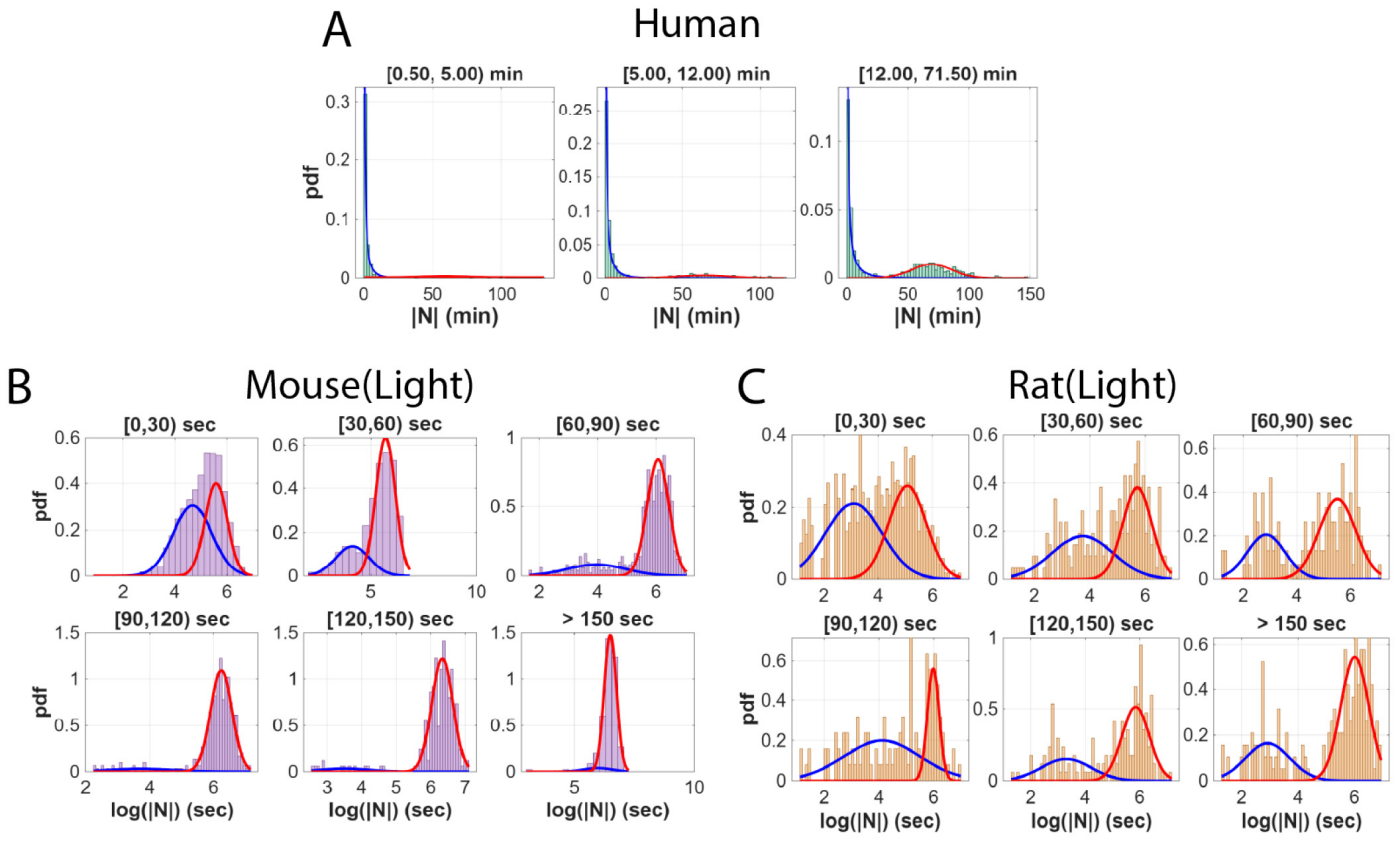
Mixture model fits of inter-REMS |*N*| distributions partitioned by |REMpre| duration. Mixture models fitted to |*N*| distributions for human data **(A)** and log(|*N*|) distributions for rodent data [**(B)**: mouse (light phase), **(C)**: rat (light phase)] for REMS cycles with similar durations of the preceding REMS bout |REMpre|; dark phase results for mouse and rat data are provided in Supplementary Figure S3. Each subpanel corresponds to a distinct |REMpre| bin (ranges in seconds or minutes shown in panel titles). Histograms show empirical pdfs, where blue and red curves denote the short- and long-interval components of the mixture model.

To characterize the statistical structure of inter-REMS durations across species, we examined the distribution of cumulative NREMS sleep in inter-REMS intervals, quantified by |*N*| (Figure 3). Despite differences in absolute timing and distributional shape across species, all three distributions span both short and long inter–REMS intervals. The human and rat data exhibit a pronounced enrichment of short |*N*| durations with a broad right tail at longer |*N*|. This pattern is consistent with earlier studies showing that inter–REM intervals cluster into two dominant time scales, corresponding to short “sequential”İand longer “single” REMS cycles (Esposito et al., 2004; Barbato and Wehr, 1998; Zamboni et al., 1999). When all REMS cycles are considered together in the mouse data, this pattern is less obvious, instead exhibiting a broad, skewed distribution across a wide range of |*N*| durations with considerable overlap between shorter and longer durations. However, following previous work (Park et al., 2021; Ginsberg et al., 2024), we partitioned REMS cycles by |REMpre| and for the rodent data considered the distribution of log(|*N*|) which revealed bimodal |*N*| distributions for all species (Figure 4).

We modeled these empirical distributions of log(|*N*|) for the rodent data and |*N*| for the human data in each |REMpre| bin using mixture models (MMs, Figure 4). For both rodent data sets, we fit a two-component Gaussian mixture model (GMM) to log(|*N*|) in fixed-width |REMpre| bins of 30 (s), namely [0, 30), [30, 60), [60, 90), [90, 150), and >150 (s) (Figures 4B, C) following previous work (Park et al., 2021; Ginsberg et al., 2024) (see Supplementary Figure S3 for rodent dark-phase results). Parameters for the fit GMM functions are listed in Supplementary Tables S1–S4. For the human data, REMS cycles were partitioned based on |REMpre| into three bins where |REMpre| = [0.5, 5), [5, 12) and [12, 71.5) min (Figure 4A), and model fits were performed on the three resulting empirical |*N*| cumulative distributions (in minutes). Because the human |*N*| distributions show a non-negligible point mass at the measurement floor *x*_min_ = 30s, corresponding to the sleep scoring epoch, together with a broad heavy right tail, we did not fit a Gaussian mixture model on log(|*N*|). Instead, we modeled the human |*N*| distributions using a three-part mixture consisting of an atom (point mass) at *x*_min_, a short-duration continuous component given by the normalized *E*1 (exponential integral function) form ∝*e*^−*rt*^/*t* on [*x*_min_, ∞), and a long-duration truncated normal component. Details of the fitting procedure including likelihood estimation, fitting constraints and bootstrap validation are given in Supplementary Section S3. Parameters for the fit three-part MM are given in Supplementary Table S12.

The MM fits provide a quantitative method to categorize sequential and single REMS cycles (Park et al., 2021; Ginsberg et al., 2024). The intersection point of the short and long model components (blue and red curves in Figure 4) determines a threshold value where REMS cycles with |*N*| less than the intersection point are considered sequential cycles and those with |*N*| greater than the intersection point are considered single cycles. In all species across |REMpre| bins, the longer-duration (“single-cycle”) model component systematically shifts to larger values with increasing |REMpre|, indicating that longer preceding REMS bouts are associated with longer subsequent |*N*| duration. This pattern is consistent with the positive association between |REMpre| and |IREM| (Figure 2). For rodent data, the shorter-duration (“sequential-cycle”) component varies comparatively little across |REMpre| bins, suggesting that the characteristic timescale of short-interval REMS cycling is relatively stable in rodent sleep.

Across species, the fitted mixture models closely tracked the empirical |*N*| distributions. Agreement was quantified using Kolmogorv–Smirnov (KS) statistics comparing empirical and fitted CDFs. For rodent data, KS-best distances for the 2-component GMM fits to log(|*N*|) were consistently small at the pooled level (*D* = 0.0157–0.0499) and remained modest across REMpre bins (typically *D*≲0.06; see Supplementary Table S5 in the Supplementary material), with the largest deviations confined to a small number of tail bins (maximum *D* = 0.0908 in Mouse (Dark) Bin 6 and *D* = 0.0884 in Rat (Dark) Bin 4). Consistent with these distances, KS diagnostic *p*-values exceeded 0.05 for every rodent dataset in all bin-specific fits (Supplementary Table S6 in the Supplementary material), indicating no bin exhibited an obvious distributional mismatch. For human data, KS distances were determined to be insignificant by refit parametric bootstrapping (see Supplementary Table S12 and Supplementary Section S3 for details).

Together, these diagnostics support the conclusion that mixture-based models provide faithful statistical summaries of inter–REMS NREMS accumulation across species and within |REMpre| bins, with the largest departures concentrated in a small number of low-sample, tail-duration bins. From these fitted distributions we compute a continuous function for the cumulative distribution function (CDF) for |*N*|, *F*(|*N*|), which serves as the foundation for calculating the REM-propensity function *P*(*t*, Δ) in the next section.

### 2.4 REMS propensity *P*(*t*, Δ) across species

Building on the MM representations of |*N*| distributions, we next compute the REMS propensity function *P*(*t*, Δ) that quantifies the probability of entering REMS in the near future as NREMS accumulates. After *t* seconds of accumulation of NREMS since the last REMS bout, the probability that a transition to REMS occurs before an additional Δ seconds of NREMS is given by

$$\begin{array}{l} P\left(|N|,\Delta \right)=\frac{F\left(|N|+\Delta \right)-F\left(\left|N\right|\right)}{1-F\left(\left|N\right|\right)}, \end{array}$$ (1)

where *F*(|*N*|) is the cumulative distribution function (CDF) of |*N*| obtained from the species-specific MM fit functions. We fixed Δ = 30 s to represent a short “near-future” window similar to prior work (Ginsberg et al., 2024). By definition, *P*(*t*, Δ) reflects the instantaneous likelihood that an ongoing NREMS episode will terminate in REMS within the next Δ = 30 s.

**Figure 5:**
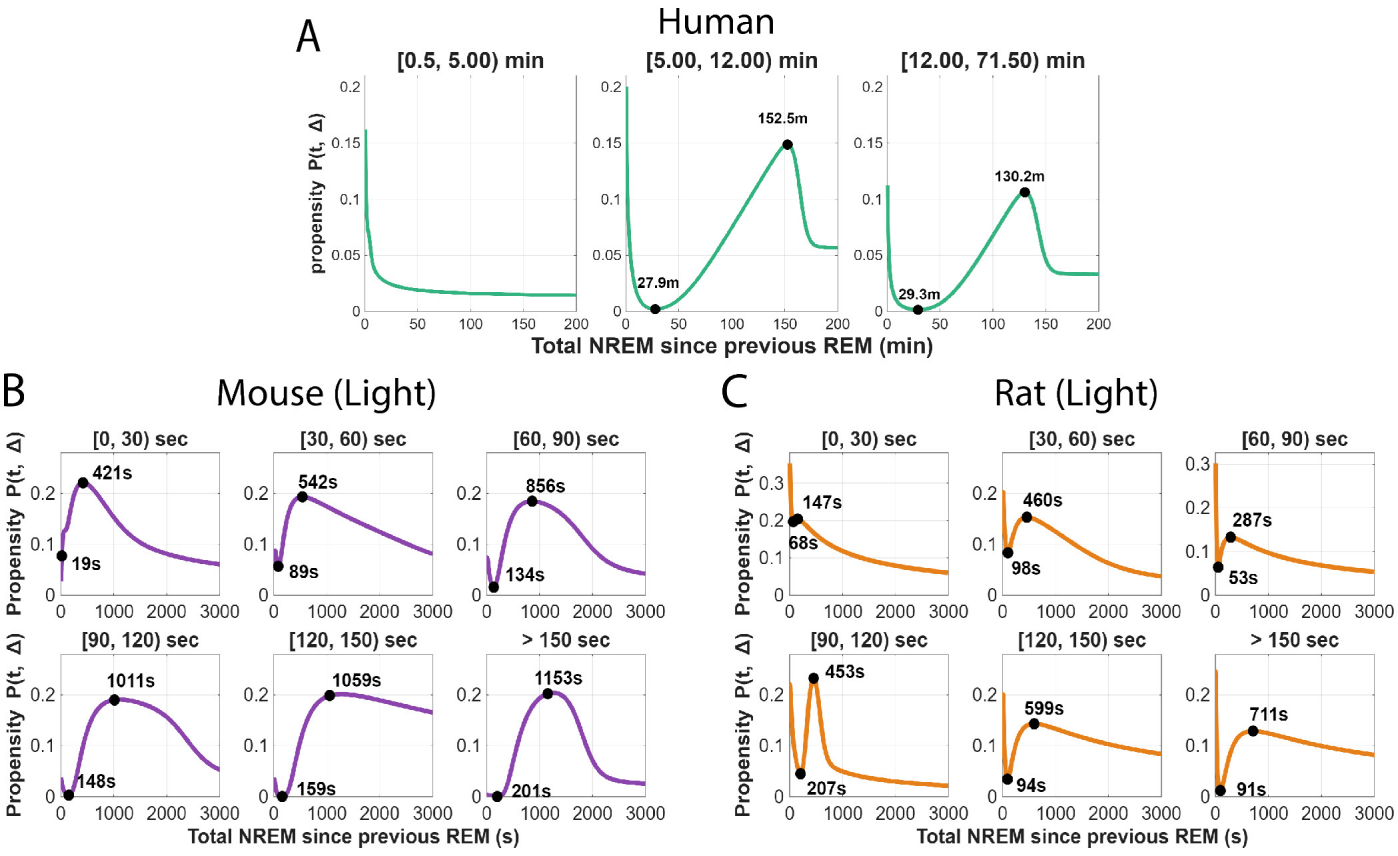
REMS propensity functions grouped by |REMpre| durations. Propensity functions *P*(*t*, Δ) (Equation 1) are shown for REMS cycles grouped by similar |REMpre| durations, corresponding to the binned inter–REMS |*N*| distributions in Figure 4, for **(A)** human, **(B)** mouse (light phase), and **(C)** rat (light phase) data. For human data, *P*(*t*, Δ) was computed from the three part atom + E1-short + truncated-normal mixture model fit to |*N*| (in minutes); for rodents, *P*(*t*, Δ) was computed from the two-component Gaussian mixture model (GMM) fit to log(|*N*|) (in seconds). In each panel, *P*(*t*, Δ) is plotted as a function of the accumulated NREMS duration *t* since the prior REMS bout. Black markers denote local minima (troughs) and maxima (peaks) with the correponding |*N*| values annotated. Dark-phase mouse and rat results are shown in Supplementary Figure S4.

Figure 5 shows the REMS propensity function curves *P*(*t*, Δ) computed from the fitted mixture models, in each |REMpre| bin, for each species using the inter–REMS |*N*| distributions in Figure 4 (mouse and rat dark phase propensity curves are shown in Supplementary Figure S4). In all species and in all but the shortest |REMpre| bins, *P*(*t*, Δ) exhibited a characteristic non-monotonic profile: after an initial transient, it increased with NREMS accumulation, reached a clear local maximum, and then decayed with further NREMS accumulation. This profile suggests that the propensity to enter REMS increases only up to a finite amount of NREMS accumulation before declining. In other words, once accumulated NREMS time becomes very long, it alone is insufficient to explain REMS timing and additional factors (e.g., circadian phase, arousal/wake intrusions, or other regulatory processes) likely contribute more strongly to the ongoing sleep pattern.

The presence of troughs (black markers) in *P*(*t*, Δ) at short values of |*N*| reflects the prevalence of sequential REMS cycles that feature minimal NREMS accumulation in the inter–REMS interval (Figure 5). This suggests that *P*(*t*, Δ) may not be applicable to predicting REMS onset in sequential cycles with |*N*| durations less than its trough value but is more applicable for REM onset prediction in longer single cycles with |*N*| durations between the trough and the peak. In the human data, for short |REMpre| durations ([0.5, 5) min), the propensity *P*(*t*, Δ) decreases rapidly with increasing accumulated NREM time and remains low thereafter, with no pronounced interior maximum, consistent with short duration REMS bouts occurring as sequential REMS cycles in which REMS re-entry typically occurs after relatively little intervening NREMS. While the profile of the propensity function in this |REMpre| bin is similar to exponential or power-law distributions, its shape is more complicated since it is computed from the three-part MM whose components are neither exponential or power-law.

The ranges of |*N*| (human) or log(|*N*|) (rodent) values at the local peaks of *P*(*t*, Δ) (black markers) vary across species (Figure 5). The longest durations occur in humans and the mice show longer durations than the rats, supporting the interpretation that *P*(*t*, Δ) encodes a species-specific homeostatic timescale for REM pressure. For the rodent propensity functions, the |*N*| locations of the peaks shift to larger values with increasing |REMpre| (with some variability for rats), and these shifts are larger than those observed for trough locations. This pattern mirrors the positive association between |REMpre| and inter–REM interval duration: longer preceding REMS bouts are followed by longer NREMS accumulation before the next REMS episode. This trend was not observed in the propensity functions computed from the human data for the two longer |REMpre| bins that exhibit a local trough and peak. The occurrence of these peaks in *P*(*t*, Δ) and the shifts in their locations with increasing |REMpre| indicates a characteristic “preferred” waiting time scale for REMS re-entry following longer REMS bouts, supporting a transition from primarily sequential cycling toward more isolated REMS episodes separated by extended NREMS accumulation.

Taken together, these results demonstrate that the temporal organization of REMS onset shares a common probabilistic structure across mammals, while the characteristic timescales and slopes of this structure are species dependent. The non-monotonic shape of *P*(*t*, Δ) is consistent with a limited-range NREMS-dependent drive that increases the likelihood of REMS transitions up to a species-specific duration, after which additional factors beyond accumulated NREMS time likely contribute to REMS timing (see Discussion).

### 2.5 Testing the predictive utility of REMS propensity *P*(*t*, Δ)

To assess whether the REMS propensity measure *P*(*t*, Δ) carries predictive information about a subsequent REMS episode and whether this relationship differs across species, we examined the association between *P*(*t*, Δ) evaluated at REMS onset and the duration of the ensuing REMS bout (|REMpost|). We focus on REM cycles whose NREMS accumulation |*N*| lies in the increasing propensity regime (between the trough and peak of *P*(*t*, Δ); see Methods). In this regime propensity varies systematically with |*N*| and therefore provides a meaningful continuous predictor.

**Figure 6:**
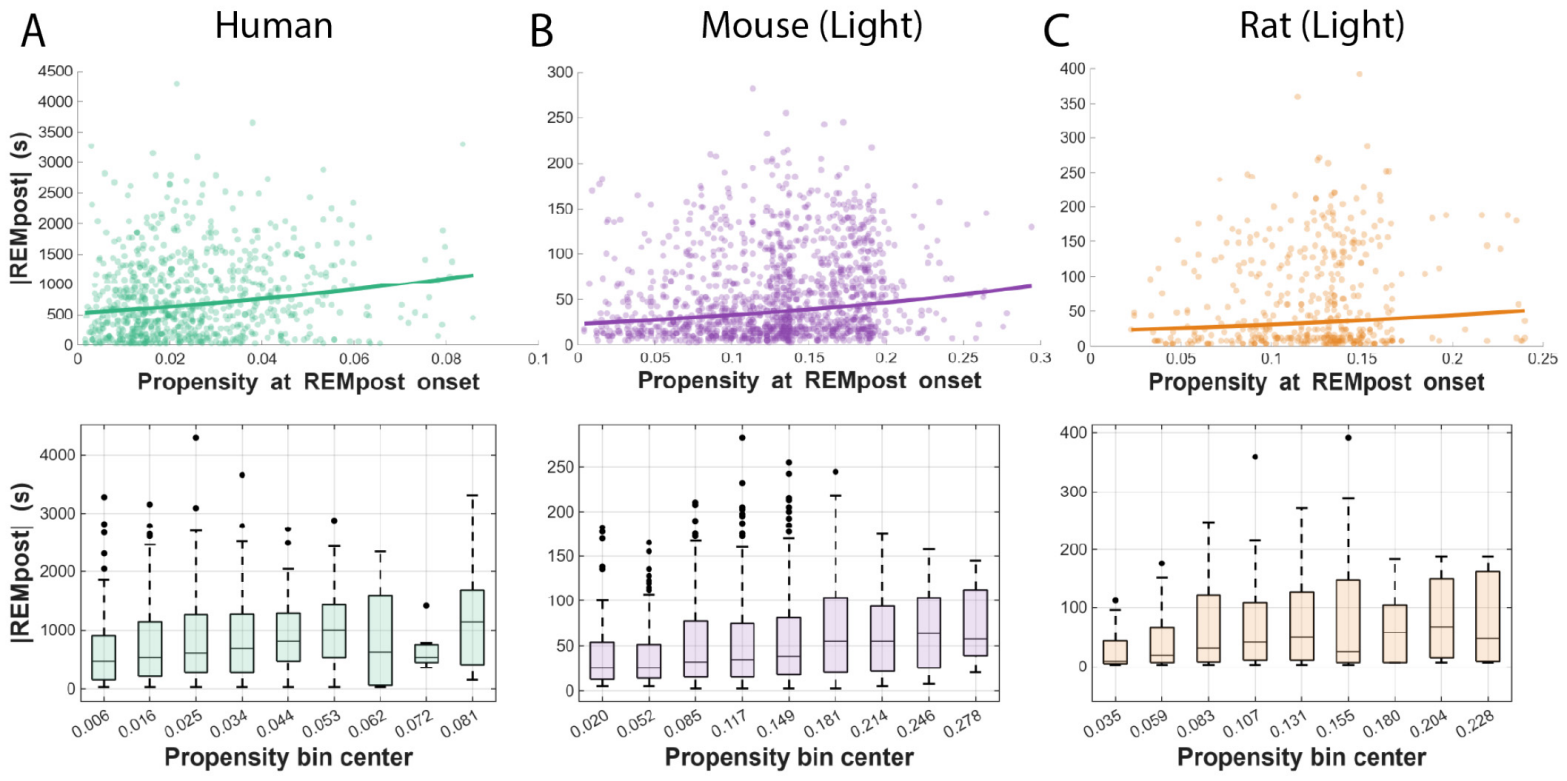
Correlation between REMS propensity and the duration of the subsequent REMS bout across species. Top row: Scatter plots show the relationship between the REMS propensity at REMS onset and the duration of the following REMS episode (|REMpost|) (in seconds) for **(A)** human, **(B)** mouse (light phase), and **(C)** rat (light phase) REMS cycles. Each point represents a single REMS cycle and solid lines denote population-level fitted trends from linear mixed-effects (LME) models computed over cycles within the increasing-propensity regime. To account for repeated REMS cycles contributed by the same subject or animal, significance was assessed using log-log linear mixed-effects models with subject/animal as a random intercept (and dataset source included as a fixed effect for human data). The estimated fixed-effect coefficient for propensity was positive and significant in all three panels: human (β = 9.195, *p* = 3.06 × 10^−4^), mouse (light) (β = 3.530, *p* = 1.71 × 10^−11^), and rat (light) (β = 3.537, *p* = 0.0298). Bottom row: Box-and-whisker summaries of |REMpost| distributions across binned propensity values for **(A)** human, **(B)** mouse (light), and **(C)** rat (light). Boxes denote the interquartile range with a median line; whiskers extend to 1.5 × IQR beyond each end of the interquartile range; points denote outliers. Full statistical analyses are reported in Supplementary Section S5.

Figure 6 summarizes this relationship across species. The top row (a–c) shows cycle-by-cycle scatter plots of |REMpost| vs. the propensity value at REMS onset for human, mouse (light phase), and rat (light phase) REMS cycles. Population-level trends were determined by linear mixed-effects models (see Supplementary Section S5 for details). In all three datasets, the fitted trends were significantly positive, indicating a consistent positive association: REMS episodes that begin at higher propensity values tend to be followed by longer REMS bouts.

As a robustness check, we also evaluated this relationship using Gamma generalized linear mixed-effects (Gamma-GLME) models with log link, which are well-suited for positive, right-skewed duration outcomes. Under this alternative model, the positive association remained significant. The bottom row provides a complementary non-parametric view where REMS cycles are binned according to the propensity value at their onset. Across species, the box-and-whisker summaries of the corresponding |REMpost| distributions show an overall upward shift in median values (lines across boxes) with increasing propensity bin values. This confirms that the positive trend is not driven by a small number of outliers, but instead reflects a broad shift in the typical (*median*) |REMpost| as propensity increases. Analogous analysis for rodent dark phase data is shown in Supplementary Figure S5 and discussed in Supplementary Section S5.

Notably, the strength and dynamic range of this relationship differ across species. Human cycles span a much wider range of |REMpost| values (including rare very long bouts), whereas rodents exhibit shorter bouts overall with tighter dispersion, consistent with their faster ultradian REMS–NREMS cycling. Nevertheless, the overall increase in both the scatter trends and binned medians indicates that *P*(*t*, Δ) captures a conserved aspect of REMS cycle organization: the state of the system at REMS entry, as summarized by REMS propensity, predicts the duration of the imminent REMS episode.

### 2.6 Temporal organization of REMS expression across the human sleep episode

Previous work has shown that REMS in humans is under strong circadian regulation and increases across the nighttime sleep episode (Czeisler et al., 1980a; Dijk and Czeisler, 1995; Dijk et al., 2010). To understand how these observations interact with the occurrence of single and sequential REMS cycles, we provide a detailed characterization of the temporal organization of REMS expression in our human data. First, we identified normalized sleep-episode deciles for each sleep record (0–100% of total time spent in sleep by the subject in increments of 10%). This normalization accounts for different total sleep durations across sleep records and allows us to determine whether REMS features, such as number of REMS bouts, REMS bout durations, sequential vs. single REMS cycles, and overall time in REMS, are uniform across the sleep episode or exhibit structured temporal gradients. Such gradients would be expected if the underlying regulatory processes governing REMS expression, including circadian REMS modulation and the homeostatic dissipation of NREMS pressure, evolve systematically over the course of the night. The following subsections demonstrate that REMS expression in humans is not temporally uniform but shows marked increases at the very beginning and at the very end of the sleep episode.

#### 2.6.1 Percent time spent in REMS increases across the night

**Figure 7:**
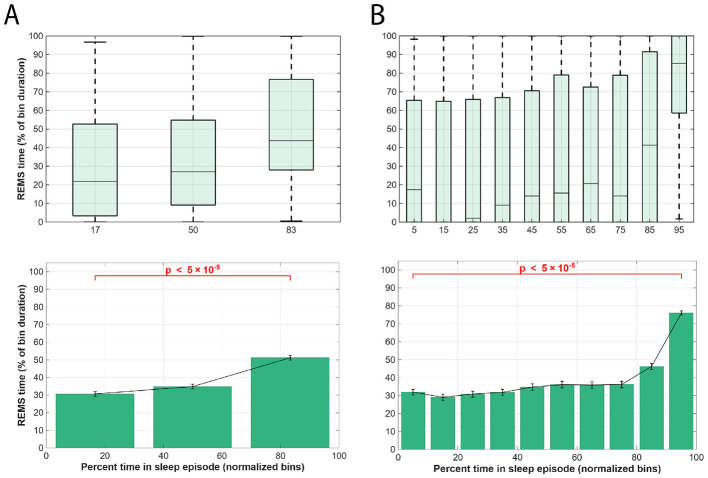
Percent time in REMS increases toward the end of the normalized sleep episode in humans. Coarse three-bin summary **(A)** and fine scale ten-bin summary **(B)** of the fraction of time spent in REMS across the normalized sleep episode after long-wake exclusion and subject-level cycle-count IQR filtering. Top: box-and-whisker plots across subjects for the subject-level REMS fraction in each normalized time bin. Bottom: bars show subject means and error bars show standard error. **(A)**: A subject-level one-sided sign-flip test comparing the last bin with the first bin showed a significant increase in REMS percentage toward the end of sleep (*p* < 5 × 10^−5^). **(B)**: The final decile exhibits a marked increase in REMS percentage relative to the beginning of sleep; a subject-level one-sided sign-flip test comparing the last decile with the first decile was highly significant (*p* < 5 × 10^−5^). In all panels, whiskers indicate the most extreme observations within 1.5 × IQR, and the REMS fraction is expressed as the percent of bin duration occupied by REMS.

Similar to previous reports in humans (Dijk et al., 2010), we find that the percent time spent in REMS increases across the sleep episode. Figure 7 shows the percent time spent in REMS when the sleep episode is divided into three (A) or ten (B) equal sized bins (bin duration varies with length of a subject's sleep episode). While the coarse three-bin representation shows a monotonic increase similar to previous results (Dijk et al., 2010), the finer ten-decile version reveals the predominance of REMS in the very late stages of the sleep episode. The box-and-whisker plots (Figure 7, top row) display the high variability in the data while the bar plots (Figure 7, bottom row) show trends in the means. While the first tenth of the sleep episode contains a similar fraction of REMS as occurs during the middle of the sleep episode, REMS fraction is not uniform in the intervening deciles. At the very end of the sleep episode, REMS percentage dominates with REMS fraction reaching up to 70% in the last decile.

This increase in REMS percent toward the end of sleep was supported statistically by subject-level one-sided sign-flip permutation tests (see Supplementary Section S4 for more details): in the three-bin analysis, the last bin showed a significant increase in REMS percentage relative to the first bin, and in the decile analysis, the final decile was likewise significantly greater than the first decile (both *p* < 5 × 10^−5^). The results indicate that the late-night predominance of REMS is a robust population-level feature, and they support the interpretation that REMS expression becomes increasingly favored as the sleep episode progresses. At the same time, the elevated REMS fraction in the first decile suggests that REMS temporal organization is not purely monotonic, but instead reflects a more structured within–night pattern with enhanced REMS expression at the beginning and end of the sleep episode.

#### 2.6.2 Number and duration of REMS bouts across the sleep episode

**Figure 8:**
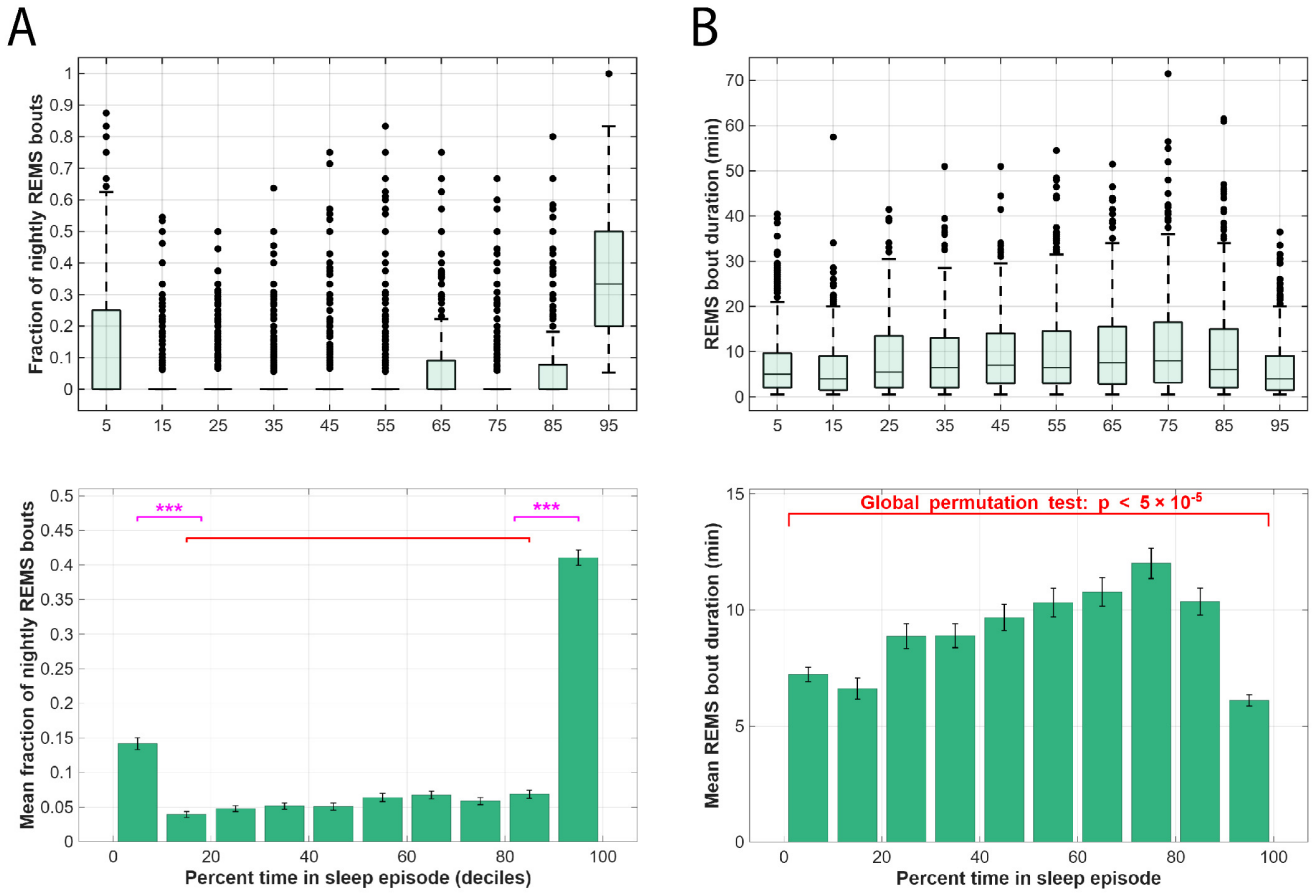
Normalized-time distribution of REMS onset frequency and REMS bout duration across the human sleep episode. Human REMS cycles were analyzed after long-wake exclusion (≥2 min) and subject-level cycle-count IQR filtering by mapping each subject's sleep episode onto a normalized time axis and dividing it into deciles. **(A)** REMS onset distribution across normalized sleep time. Top: box-and-whisker plots show the subject-level distribution of the fraction of nightly REMS bouts whose onsets occur in each decile. Bottom: bars show subject means and error bars show standard error across subjects. REMS onsets were strongly enriched near the beginning and end of the sleep episode, especially in the final decile. The red bracket denotes the middle deciles (10–90% of normalized sleep time), and the magenta asterisks indicate significant edge-vs.-middle comparisons based on subject-level paired sign-flip permutation tests: the first decile differed significantly from the middle deciles (*p* < 5 × 10^−5^), and the last decile also differed significantly from the middle deciles (*p* < 5 × 10^−5^). **(B)** REMS bout duration as a function of normalized onset decile. Top: box-and-whisker plots show the pooled distribution of REMS bout durations for bouts whose onsets fell in each decile. Bottom: bars show pooled mean REMS bout duration in each decile and error bars show standard error across bouts. REMS bout duration varied significantly across onset deciles, with the longest bouts occurring in the later-middle portion of the sleep episode and shorter bouts at the beginning and end, particularly in the final decile. The red bracket indicates the global across-decile comparison, and a within-subject label permutation test confirmed a significant dependence of REMS bout duration on onset decile (*p* < 5 × 10^−5^). In all box-and-whisker plots, whiskers extend to the most extreme observations within 1.5 × IQR, black markers indicate outliers.

To further quantify how REMS varies across the sleep episode, Figure 8 shows the mean fraction of nightly REMS bouts whose onsets occur in each normalized-time decile (A) and the mean REMS bout duration (min) for bouts initiated in each decile (B). Across subjects, REMS onset events were strongly concentrated near the beginning and end of the sleep episode: approximately 15% of nightly REMS bouts were initiated in the first decile, whereas about 41% were initiated in the last decile. Subject-level paired sign-flip permutation tests confirmed that the first decile, the last decile, and the average of the two edge deciles each differed significantly from the middle deciles (10–90% of normalized sleep time; all *p* < 5 × 10^−5^; see Supplementary Section S4 for details of the hypothesis tests).

REMS bout duration also varied significantly across onset deciles (within-subject label permutation test, *p* < 5 × 10^−5^; see Supplementary Section S4), but its pattern was not monotone. REMS bout durations were relatively short in the earliest and latest deciles, increased across the middle portion of the sleep episode, and reached their largest values in the later-middle deciles before declining again near the end of sleep. Thus, the elevated REMS percentages near the end of the sleep episode shown in Figure 7 appear to be driven by increasing REMS duration until the very end of the sleep episode when shorter, more frequent REMS bouts occur.

#### 2.6.3 Single and sequential REMS cycles exhibit distinct temporal profiles

To further dissect the temporal structure of REMS architecture, we examined the organization of REMS into single and sequential cycles as a function of REMS onset time within the normalized sleep episode. As shown above (Figure 8), the distribution of REMS onsets across the night is highly non-uniform with a substantial fraction of nightly REMS bouts occurring early and late in sleep. Here, we ask how this overall temporal distribution decomposes into single vs. sequential REMS organization, and whether these two REMS modes differ in their temporal evolution.

**Figure 9:**
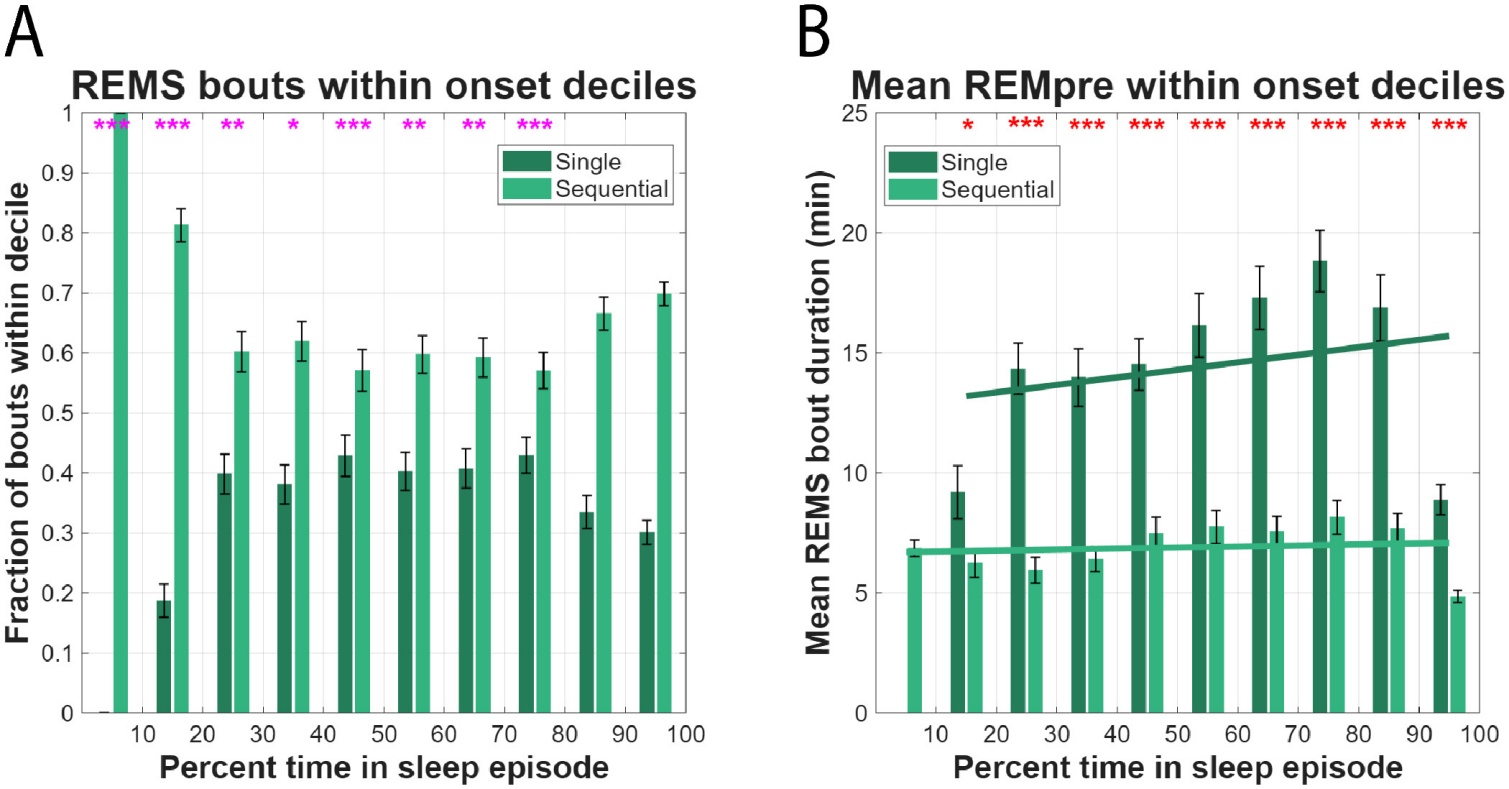
Single vs. sequential REMS cycle occurrence across the sleep episode. REMS cycles were classified as single and sequential. Each cycle was assigned to a decile based on REMS onset time within a normalized sleep episode.**(A)** Pooled fraction of REMS bouts with onset in each decile that initiate single (dark green) or sequential (light green) REMS cycles. Error bars denote binomial standard errors. Magenta asterisks indicate deciles in which the fraction of single REMS cycles differs significantly from the overall pooled baseline (two-sided exact binomial test, false discovery rate corrected), with one, two, and three asterisks denoting *p*_FDR_ < 0.05, *p*_FDR_ < 0.01, and *p*_FDR_ < 0.001, respectively. **(B)** Pooled mean REMS bout duration (|REMpre|, min) within each onset decile in single (dark green) or sequential (light green) REMS cycles. Error bars denote standard errors of the mean. Red asterisks indicate deciles with significant differences in REMS bout duration between single and sequential cycles (Welch two-sample *t*-test, false discovery rate corrected), using the same significance thresholds. Global within-subject permutation tests further showed that REMS bout duration depended significantly on onset decile for both single cycles (*p* < 5 × 10^−5^) and sequential cycles (*p* = 0.0115). The fitted trend lines further show these distinct temporal patterns: mean duration of REMS bouts in single cycles shows a clear positive trend across onset deciles, increasing from early to later portions of the sleep episode, whereas mean duration of REMS bouts in sequential cycles remains comparatively flat, showing only a weak change across deciles.

Figure 9A shows the composition of REMS cycles within each REMS-onset decile, expressed as the pooled fraction of cycles classified as single or sequential. Consistent with the distribution of REMS onsets across the sleep episode (Figure 8A), early REMS expression is qualitatively distinct: among the REMS cycles initiated in the first decile, the overwhelming majority are classified as sequential rather than single. This bias indicates that early REMS onsets predominantly occur in clustered REMS–NREMS–REMS sequences separated with short inter-REMS intervals. As the sleep episode progresses into mid-sleep, the balance shifts significantly, with single REMS cycles contributing more to REMS expression, reflecting longer inter-REMS accumulation between REMS bouts. At the very end of the sleep episode, the occurrence of sequential REMS cycles increases again, indicating a partial re-emergence of interrupted REMS organization in late sleep.

Figure 9B shows the mean REMS bout duration (|REMpre|) for single and sequential cycles within each REMS-onset decile. Across the sleep episode, REMS bouts in single cycles tend to exhibit longer mean durations than REMS bouts in sequential cycles, indicating that these two REMS cycling regimes differ not only in their temporal organization but also in REMS bout length. This separation is statistically supported by two-sample Welch t-tests comparing |REMpre| between single and sequential cycles within each decile: from the second decile onward (approximately 20–90% of the sleep episode), REMS bouts in single cycles are significantly longer than in sequential cycles after false discovery rate correction (*p*_FDR_ < 0.05). No significant difference was detected in the earliest decile, where single REMS cycles are rare.

Beyond this categorical distinction, REMS bouts in single cycles exhibit a pronounced modulation across the sleep episode, increasing in duration from early to mid-sleep and reaching their longest durations in the latter half of the sleep episode. In contrast, REMS bouts in sequential cycles remain comparatively short throughout the sleep episode, showing only modest lengthening toward the end of sleep.

This analysis of normalized sleep-time deciles shows that REMS expression in humans is strongly time-dependent across the sleep episode. The fraction of time spent in REMS increases toward the end of sleep, with the most pronounced rise occurring in the final decile, where REMS occupied the largest share of bin time (Figure 7), indicating that REMS becomes progressively more dominant as the night advances. REMS bout onsets were also highly non-uniform across the episode, being enriched near both the beginning and end of sleep and especially in the final decile (Figure 8A), suggesting that late REMS predominance is driven not only by increased REMS occupancy but also by a greater likelihood of entering REMS near the end of the sleep episode. In contrast, REMS bout duration did not increase monotonically across the night: bouts were shorter at the beginning and end of the sleep episode and longest in the later-middle deciles (Figure 8B). This indicates that the large REMS fraction late in sleep is not simply due to longer REMS bouts, but instead reflects more frequent REMS initiation, with the final part of sleep characterized by shorter but more frequent REMS episodes. Decomposing REMS expression into sequential and single cycles revealed distinct temporal structure: early REMS was dominated by sequential cycles, mid-sleep REMS was dominated by single cycles, and late sleep showed a renewed increase in sequential REMS cycle occurrence (Figure 9A). This pattern suggests that REMS organization changes systematically across the night, with early and late sleep showing more interrupted or clustered REMS expression, and mid-sleep showing more consolidated REMS expression separated by longer time in NREMS. Across most onset deciles, REMS bouts in single cycles were significantly longer than the bouts in sequential cycles (Figure 9B), indicating that temporal changes in REMS timing across the sleep episode are accompanied by systematic shifts in REMS micro-architecture. Together, these findings may reflect an evolving interaction between homeostatic and circadian influences: early in the night, strong homeostatic NREMS pressure may limit REMS consolidation, whereas later in the night, dissipation of NREMS pressure together with rising circadian REMS drive may promote frequent REMS initiation and a distinct late-sleep REMS organization (see Discussion).

## 3 Discussion

In this study, we analyzed human, rat, and mouse ultradian NREMS-REMS cycle data using the same methodology and found that the ultradian cycle structures showed many similarities among the three species despite differences in time scales and diurnal vs. nocturnal behavior. In all three species, REMS bout durations were positively correlated with the subsequent inter-REMS interval duration such that longer REMS bouts were followed by longer inter-REMS intervals. This result supports the hypothesis of an hourglass-type homeostatic process driving REMS sleep in which a longer REMS episode discharges a greater portion of accumulated REMS pressure, thereby delaying the onset of the next REMS episode (Benington and Heller, 1994b,a). Furthermore, this result is not inconsistent with the hypothesis of a post-REMS refractory period which occurs after REMS and inhibits the initiation of a subsequent REMS bout for a period of time proportional to the REMS episode (Le Bon, 2021).

Distributions of the duration of NREMS in inter-REMS intervals (|*N*|) were right-skewed for all 3 species. The human data showed a bimodal profile as previously reported (Zamboni et al., 1999; Esposito et al., 2003, 2004) and was fit well with a three-part mixture model that accounted for the high density at the lowest scoring epoch of 30 s, an initially slowly decaying tail and an approximately normal distribution at longer durations. In the rodent data, bimodality was more clearly revealed in the log(|*N*|) distribution and when REMS cycles were partitioned by REMpre duration consistent with previous work (Park et al., 2021; Ginsberg et al., 2024). For both mouse and rat data, these distibutions were fit well with two-component Gaussian mixture models. The short- and long-components of these mixture models correspond to sequential and single REMS cycles, respectively, and the intersection between these components provides a quantitative method to identify the threshold separating sequential and single REMS cycles. In both mouse and rat data, there was less separation between sequential and single REMS cycles for the shortest |REMpre| bins, reflecting that the majority of those inter-REMS intervals were short. For the longer |REMpre| bins, the long model component generally shifted to longer |*N*| values with increasing |REMpre| as predicted by the positive correlation between durations of REMS bouts and the subsequent inter-REMS interval.

Based on these mixture model fits for |*N*| (human) or log(|*N*|) (rodent) distributions, we computed REMS propensity, *P*(*t*, Δ), as a function of time *t* spent in NREMS during the inter–REMS interval. Although different mixture models were used for different species, we found that REMS propensity functions had similar profiles across species, with minimum propensity values occurring for shorter |*N*| and increasing to a local peak as |*N*| increased. Then, the propensity decreased for the remaining longer values of |*N*|. In the rodent data, the |*N*| value at propensity peak increased for longer durations of |REMpre| (albeit with some variability in this trend for the rat propensity), reflecting that longer REMS bouts are followed by longer inter-REMS intervals. This trend was not apparent in the human REMS propensity, but this may be due to only considering a small number of |REMpre|b~ins.

Importantly, the REMS propensity measure at REMS onset predicted REMS bout duration in all 3 species we considered. Specifically, in humans and rats, REMS bout duration was positively correlated with REMS propensity at REMS bout onset, consistent with previous work in mice (Ginsberg et al., 2024). This finding highlights the predictive value of the REMS propensity measure, *P*(*t*, Δ), to represent REMS pressure based on the amount of accumulated NREMS since the previous REMS bout. Furthermore, it provides a mechanism to begin understanding both the timing and duration of REMS episodes.

In our analysis, we did not distinguish different stages of NREMS in humans, namely stages 1, 2 and 3 where stage 3 is considered a deeper stage of NREMS and transitions to REMS typically occur from stage 2. While these stages are routinely scored in human sleep, they are not usually scored in rodent sleep and our rodent data did not include the separate stages. Additionally, the rodent sleep data did not separately score the transitional state pre-REMS or “intermediate stage” that can precede the transition into REMS from NREMS (Glin et al., 1991; Mandile et al., 1996).

We chose to combine all NREMS stages scored in the human data sets into one NREMS state so that the same methodology could be applied in all three species. Further analyses of appropriately scored data are necessary to identify if the evolution of REMS pressure may differ in the different NREMS stages or in the pre-REMS stage.

While short REMS bouts and short inter-REMS intervals are a widely acknowledged feature of rodent polyphasic sleep, standard sleep scoring practices for human data have tended to ignore short interruptions of sleep states in favor of scoring consolidated bouts of NREMS and REMS as they alternate across the night (Charles, 1980; Merica and Gaillard, 1991). However, raw EEG recordings of human sleep indicate a more complex story, and this scoring practice may mask similarities in the temporal architecture of sleep in different species. Although NREMS and REMS typically alternate on a 90 min ultradian timescale in human sleep, the“REM sleep” part of the cycle may include either longer, consolidated REMS bouts or shorter, fragmented bouts of REMS interspersed with NREMS. When scored without criteria to promote consolidation, the alternation between periods of majority REMS and majority NREMS on a 90 min timescale is preserved, but many shorter REMS-NREMS cycles are observed. As we seek to further our understanding of the mechanisms driving REMS-NREMS ultradian cycling, these fine details of sleep architecture may be important considerations.

Previous work to distinguish REMS cycles based on the duration of associated inter-REMS intervals was quantitatively formalized in rats as single (one longer bout) or sequential (multiple shorter bouts close together) REMS cycles (Zamboni et al., 1999). These dynamics have also been observed in human sleep data (Merica and Gaillard, 1991; Esposito et al., 2003), however, to our knowledge, they have not been quantitatively formalized beyond being used to designate an arbitrary threshold for inter-REMS intervals (Merica and Gaillard, 1991). We formalized this analysis for a heterogeneous collection of human data representing participants from a range of demographic groups, collected over different time periods, with different inclusion/exclusion criteria, and with different experimental and scoring protocols. Despite the heterogeneity, a clear bimodal distribution in inter-REMS interval duration was observed in the pooled data. The inter-REMS NREMS duration (|*N*|) data were fit with a mixture model that, although it had different components compared to the mixture model for the rodents, provides a data-driven, quantitative method for differentiating sequential and single REMS cycles and computing REMS propensity.

Furthermore, our analysis of the temporal organization of REMS bouts across the human sleep episode helps to better understand how single and sequential REMS cycles contribute to the well-documented increase in REMS across the sleep episode in humans. By considering REMS bouts within deciles of the normalized sleep episode, we found that a higher fraction of REMS bouts occurs within the first and last deciles of the sleep episode. Interestingly, the majority of REMS cycles in the first decile were sequential while there was a higher proportion of single REMS cycles during mid-sleep. During the last deciles, sequential cycles again occurred more often than single cycles. This suggests that at the beginning and end of a sleep episode, REMS bouts are more likely to be fragmented. This fragmentation may reflect effects of NREMS homeostasis and circadian modulation of REMS. For example, early in the sleep episode, the predominant occurrence of sequential cycles with short REM bouts may reflect unstable REMS expression under strong homeostatic NREMS pressure. During mid-sleep, REMS bouts become longer and more consolidated leading to dominance of single REMS organization with a lower fraction of REMS onsets, potentially caused by decreasing NREMS pressure and increasing circadian REMS drive. Late in the sleep episode, when the percent time spent in REMS is the highest, the reappearance of sequential REMS organization and the eventual decrease in REMS bout durations may reflect high circadian REMS drive expressed in the absence of high NREMS pressure.

Our findings demonstrate that changes in REMS timing across sleep episodes in humans are accompanied by systematic shifts in REMS micro-architecture revealing distinct temporal regimes of REMS organization likely governed by evolving homeostatic and circadian influences. Future work utilizing controlled experiments with standardized scoring practices that allow for fragmentation of states are needed to understand this dynamic process. Furthermore, such studies may enable the investigation of age, sex, or disease effects on single and sequential REMS cycles as well as ultradian cycling.

As experimental studies continue to investigate the neural regions and processes that govern ultradian NREMS-REMS alternation in rodent models, it is important to account for how those processes influence single and sequential REMS cycles. Moreover, as we work to infer whether the same neural processes govern human ultradian cycling, it will be important to understand the temporal architecture of single and sequential REMS cycles in humans. Our analysis identifies clear similarities in human and rodent ultradian cycling and REMS regulation while also formalizing some potential differences. Future work in these and other species is needed to identify the significance of single and sequential REMS cycles and the role of circadian modulation and homeostatic pressure for both REMS and NREMS in producing these cycles.

## 4 Methods

### 4.1 Sleep recordings

#### 4.1.1 Human

Human sleep recordings (573 total recordings) from subjects without serious health or sleep conditions were collected from 3 public online databases containing polysomnographic (PSG) recordings: (1) the Sleep-EDF Database Expanded on Physionet (https://www.physionet.org/content/sleep-edfx/1.0.0/), (2) Mignot Nature Communications dataset supported by the National Sleep Research Resource (https://sleepdata.org/datasets/mnc), and (3) Bitbrain Open Access Sleep (BOAS) dataset (https://www.bitbrain.com/science/eeg-datasets).

From the Sleep-EDF Database Expanded, we used data from 152 subjects in the Sleep Cassette Study which includes PSG sleep records from healthy Caucasians (54% female) aged 25–101 who were not taking any sleep-related medication (Kemp et al., 2000; Goldberger et al., 2000). Each participant (except 3) underwent two subsequent nights of recordings in their own homes. This resulted in 153 recordings from 78 unique participants. We used the hypnogram files that contained annotations of sleep patterns corresponding to the PSG in 30-s epochs. The Sleep-EDF dataset was collected from 1987-1991 and scored based on the Rechtschaffen and Kales (R & K) scoring criteria (Rechtschaffen et al., 1968). Scored states were wake, REM sleep, NREM states 1, 2, 3, and 4, and movement. We renamed all NREM sleep states as a single NREM state.

From the Mignot Nature Communications (MNC) dataset, we used data from 294 control (non-narcoleptic) subjects from three of the study cohorts (SSC, CNC, and DHC) (Zhang et al., 2018; Stephansen et al., 2018). From the MNC dataset, we selected three study cohorts: the Stanford Sleep Cohort (SSC) (Andlauer et al., 2013; Moore IV et al., 2014), the Chinese Narcolepsy Cohort (CNC) (Andlauer et al., 2013), and the Danish Hypersomnia Cohort (DHC) (Christensen et al., 2017). The SSC cohort recordings were scored using the R & K critera (Moore IV et al., 2014; Rechtschaffen et al., 1968). The PSG recording of the DHC participants were scored by experienced technicians using the American Academy of Sleep Medicine (AASM) criteria (Berry et al., 2015). The data in these cohorts was annotated in 30-s epochs as wake, REM sleep, NREM sleep states 1, 2 and 3, and unscored. We renamed all NREM sleep states as a single NREM state.

The BOAS dataset contains PSG recordings from participants who ranged in age (18–82 years old), sex (59% female), and body mass index (18.3–33.3) (Lopez-Larraz et al., 2024). There were 108 unique individuals who participated in the study. Sleep staging was scored by three experts who independently scored the data following criteria developed by the American Academy of Sleep Medicine (AASM) (Berry et al., 2015). Then, a consensus label was determined (Lopez-Larraz et al., 2024). Exclusion criteria only included severe conditions that may have affected the feasibility or safety of the protocol. We used data from 127 recordings that were manually annotated in 30-s epochs as wake, REM sleep, NREM sleep states 1, 2 and 3. We renamed all NREM sleep states as a single NREM state.

**Figure 10:**
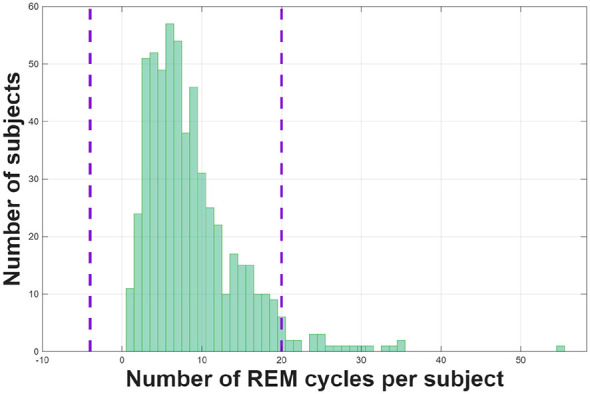
Subject-level distribution of retained REMS cycle counts in the human dataset after application of the long-wake filtering criterion. Dashed vertical lines denote the quartile-based outlier bounds, *Q*_1_−1.5IQR and *Q*_3_+1.5IQR, used to remove subjects with atypical numbers of analyzable REMS cycles. This subject-level filtering step was applied before human mixture-model fitting.

Because the human dataset was assembled from multiple public cohorts and included recordings of varying duration and structure, we applied an additional subject-level outlier filter to limit disproportionate influence from subjects contributing an unusually small or unusually large number of analyzable REMS cycles (Figure 10). After applying the long-wake (LW) exclusion criterion (see below) and retaining only valid REMS cycles, we counted the number of remaining cycles contributed by each subject. Letting *Q*_1_ and *Q*_3_ denote the first and third quartiles of the subject-wise cycle counts, and IQR = *Q*_3_−*Q*_1_, we classified as outliers any subjects with cycle counts below *Q*_1_−1.5IQR or above *Q*_3_+1.5IQR. All REMS cycles from such subjects were excluded from our analysis.

After this filtering step, 515 of the 573 human recordings remained for analysis.

#### 4.1.2 Rat

Rat sleep data were collected from control condition experiments from (Silverstein et al. 2024). Briefly, adult Sprague Dawley rats (*n* = 5 rats (4 female, 1 male), 300–350 g, aged 8–12 weeks, Charles River Inc., Wilmington, MA) maintained on a 12:12 light:dark cycle (lights on at 8:00 a.m.) and with *ad libitum* food and water, were used for all recordings. The experimental protocol was approved by the Institutional Animal Care and Use Committee at the University of Michigan, Ann Arbor, and was conducted in compliance with the Guide for the Care and Use of Laboratory Animals (Ed 8, National Academies Press) and ARRIVE Guidelines. Under isoflurane anesthesia, stainless steel screw electrodes were implanted across the cortex for electroencephalogram (EEG) recordings and wires were positioned in the dorsal nuchal muscles to record electromyogram (EMG). Monopolar EEG (0.1–300 Hz, sampling rate 1 kHz) was recorded in a bipolar montage (0.1-125 Hz, sampling rate 500 Hz) for polysomnography (frontal-frontal, frontal-parietal, and parietal-parietal). The EMG was bandpass filtered between 0.1 Hz and 125 Hz and sampled at 500 Hz. Bipolar EEG and EMG data over a 48-h period were manually scored (SleepSign; Kissei Comtec Inc., Matsumoto, Japan) in 4-s epochs into (1) wake state: low-amplitude fast EEG along with high muscle tone, (2) NREMS: high-amplitude slow EEG along with low muscle tone, and (3) REMS: low-amplitude fast EEG along with muscle atonia.

#### 4.1.3 Mouse

Mouse sleep data were collected as described in Park et al. (2021). Briefly, male or female C57BL/6J mice (Jackson Laboratory stock no. 000664) housed on a 12-h dark/12-h light cycle (lights on between 7 a.m. and 7 p.m.), aged 6–12 weeks with *ad libitum* access to food and water were used for all recordings. All experimental procedures were approved by the Institutional Animal Care and Use Committee (IACUC) at the University of Pennsylvania and conducted in accordance with the National Institutes of Health Office of Laboratory Animal Welfare Policy. Under isoflurane anesthesia, stainless steel screw electrodes were implanted over the parietal and prefrontal cortex and the cerebellum for EEG recordings, and wires were inserted into the neck muscle for EMG recording. EEG and EMG signals, recorded using an RHD2000 amplifier (Intan Technologies, Los Angeles, CA, United States, sampling rate 1 kHz), were processed by custom software in 2.5-s epochs into (1) wake state: low delta (0.5-4 Hz) power and/or high gamma (100–150 Hz) and high EMG power; (2) NREMS: high delta power,low theta (5–12 Hz) /delta power ratio and low EMG power; and (3) REMS: high theta/delta power ratio, low EMG power, and low delta power. All recordings were then manually rescored to verify classification. Data are available online at https://zenodo.org/records/5817119#.YdvvNC_kGTc and https://zenodo.org/records/5820559#.Ydvvcy_kGTc.

### 4.2 Computation of REMS propensity

REMS propensity is quantified as a predictive conditional probability of entering REMS based on the local sleep history preceding each REMS bout. Our analysis builds on the probabilistic framework introduced in previous work (Park et al., 2021; Ginsberg et al., 2024) and we summarize the key steps here.

#### 4.2.1 REMS cycle definition, |REMpre|, |REMpost|, and |REMpre| binning

Sleep was partitioned into REMS cycles, defined as the interval from the onset of one REMS bout to the onset of the next. Each cycle is characterized by the duration of its initial REMS bout (|REMpre|), followed by an inter-REMS interval of duration |IREM| with cumulative time spent in NREMS sleep |*N*|. The REMS bout terminating the cycle is referred to as REMpost and represents the subsequent expression of REMS following the inter-REMS interval. Thus, each REMS cycle captures the relationship between an initial REMS episode (|REMpre|), the intervening inter-REMS interval, and the timing and duration of the next REMS bout (|REMpost|).

#### 4.2.2 Long wake filtering and statistical validation

To ensure that REMS propensity reflects intrinsic inter-REMS dynamics during sleep behavior, we excluded REMS cycles containing prolonged wake episodes within the inter-REMS interval. The choice of the threshold defining a long wake (LW) episode was statistically validated using two-sample Kolmogorov-Smirnov (KS) tests and Bayesian Information Critierion (BIC) tests (see Supplementary Section S2). Specifically, for candidate wake duration thresholds, REMS cycles were separated into those with and without wake bouts exceeding the threshold, and the resulting distributions of cumulative inter-REMS |*N*| quantities were compared. KS and BIC tests revealed more stable, well-structured inter-REMS |*N*| distributions when cycles containing sufficiently long wake episodes were removed. Based on this validation, an LW threshold of ≥2 min was used for mouse, rat and human sleep.

#### 4.2.3 Mixture model fitting and goodness-of-fit

REMS cycles were partitioned into predefined |REMpre| bins of 30 s intervals for rodent data and into 3 bins with equal numbers of REMS cycles for human data. For REMS cycles in each |REMpre| bin, the distributions of cumulative NREMS duration in the inter-REMS interval (|*N*|) were fit with mixture models. For rodent data, distributions of log(|*N*|) were fit with Gaussian mixture models (GMMs) as in previous work (Park et al., 2021; Ginsberg et al., 2024). For human data, |*N*| distributions were fit with a three-part mixture model consisting of an atom ( point mass) at *x*_min_, a short-duration continuous component given by the normalized *E*1 form ∝*e*^−*rt*^/*t* on [*x*_min_, ∞), and a long-duration truncated normal component (see Supplementary Section S3). Model adequacy was assessed using corrected Kolmogorov-Smirnov tests to ensure consistency between the fitted models and empirical distributions (for rodent data, see Supplementary Tables S5, S6; for human data, see Supplementary Section S3).

#### 4.2.4 Definition of single and sequential REMS bouts

**Figure 11:**
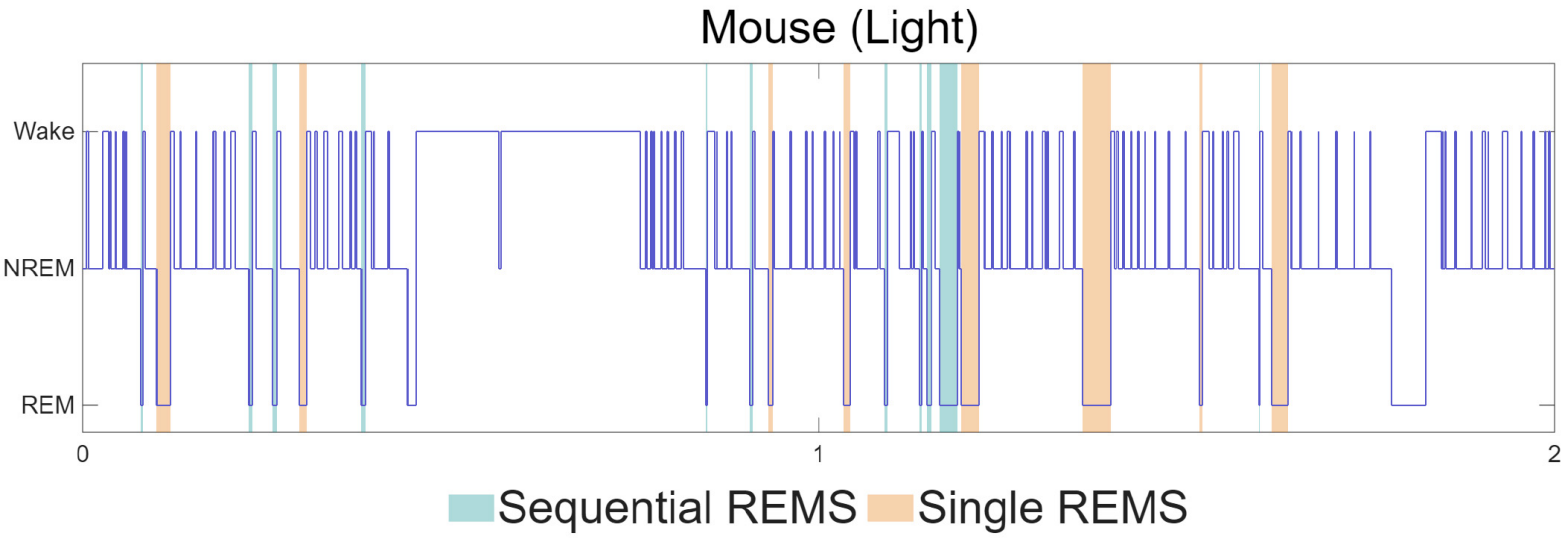
Example mouse hypnogram in the light phase illustrating sequential and single REMS cycles. A representative 2-h segment of a mouse hypnogram recorded during the light phase is shown, with vigilance states labeled as Wake, NREMS, and REMS. Colored vertical bands indicate REMS bouts classified as initiating a sequential (teal) or single (orange) REMS cycle. The classification is made according to the NREM accumulation |*N*| in the subsequent inter-REMS interval, using REMpre-bin-specific cutoff values derived from the GMM fits of the mouse population data in the light phase. The long-wake (LW) exclusion criteria was applied to the REMS cycles. REMS bouts without a colored vertical band were excluded from the analysis because they did not satisfy the long-wake filter, and the final REMS bout in the segment is not shown as a full cycle because the subsequent cycle is incomplete.

In the mixture model fits of the distributions of inter-REMS NREMS accumulation |*N*| in each |REMpre| bin, the two mixture components correspond to a short-|*N*| mode and a long-|*N*| mode. We defined the boundary between regimes as the intersection point of the two weighted component density curves, i.e., the value of |*N*| at which the probabilities of belonging to the short and long modes are equal. Cycles with |*N*| below the intersection (more likely to belong to the short-|*N*| mode) were classified as sequential REMS cycles, reflecting REMS cycles with short inter-REMS NREMS duration. Cycles with |*N*| above the intersection (more likely to belong to the long-|*N*| mode) were classified as single REMS cycles, corresponding to REMS episodes followed by more substantial NREMS accumulation before the next REMS episode. Figure 11 shows an example hypnogram for mouse sleep in the light phase where REMS cycles are labeled as single or sequential.

#### 4.2.5 REMS propensity function

Following Ginsberg et al. (2024), REMS propensity was defined as the conditional probability that a transition into REMS occurs before an additional fixed increment of NREMS accumulates. This quantity can be interpreted as a discrete time hazard-like function derived from the cumulative distribution function of inter-REMS NREMS duration |*N*|. Propensity functions were computed within each |REMpre| bin and evaluated at REMS onset, yielding a predictive measure of local REMS sleep pressure.

### 4.3 Analysis of the association between |REMpre| and |IREM|

To assess the relationship between the duration of a REMS bout and the duration of the subsequent inter-REMS interval while accounting for repeated REMS cycles contributed by the same subject or animal, we used mixed-effects models. For the human data, we fit a log-log linear mixed-effects model with log(|IREM|) as the response, log(|REMpre|) as the predictor, dataset source as a fixed effect, and subject as a random intercept. For the rodent data, analogous log-log linear mixed-effects models were fit separately for each species and light/dark condition, with log(|IREM|) as the response, log(|REMpre|) as the predictor, and animal as a random intercept. As a robustness check, we additionally fit Gamma generalized linear mixed-effects models with log link, using |IREM| as the response and the same fixed- and random-effect structure. As a further sensitivity analysis, we computed subject-/animal-level median values of |REMpre| and |IREM| and evaluated their association using Spearman correlation. For all analyses, REMS cycles were first filtered using the same long-wake exclusion criterion described above. See Supplementary Section S5 for details.

### 4.4 Analysis of REMS expression across the normalized human sleep episode

To characterize how REMS expression varies across the human sleep episode, we analyzed the temporal distribution of REMS in the human data after applying the LW exclusion criterion (see above) and a subject-level outlier filter based on the number of valid REMS cycles exhibited per subject after LW filtering. Sleep records with cycle counts outside the interquartile-range criterion (< *Q*_1_−1.5IQR or >*Q*_3_+1.5IQR) were excluded to reduce disproportionate influence from outliers. Only retained REMS cycles were used in our analyses.

For each subject, the duration of the complete sleep episode was mapped onto a normalized time axis spanning 0–100% which was then partitioned into ten equal deciles. The normalized time of onset for each of the subject's retained REMS bouts was then computed by mapping REMS episode onset times from the start of the sleep episode onto the normalized time axis. Normalized REMS bout onset times were then assigned to their appropriate deciles.

After normalizing REMS bout onset times for all subjects, we then quantified three complementary features of REMS expression across normalized time in sleep. First, to measure REMS onset distribution, we computed for each subject the fraction of REMS bouts whose onsets occurred in each decile. Second, to quantify the distribution of REMS time across the sleep episode, we calculated for each subject the fraction of total REMS time falling within each decile. For this analysis, REMS time was assigned to deciles according to its temporal overlap with each decile bin. Third, to determine how REMS bout duration depended on its onset time in the sleep episode, for each decile we computed the mean durations of REMS bouts whose onset occurred in that decile, regardless of whether the REMS bout duration temporally overlapped with neighboring deciles.

For the REMS onset-distribution and REMS-time analyses, subject-level fractions were first computed within each subject and then averaged across subjects. For the bout-duration analysis, descriptive plots show pooled bout-level means by decile, whereas statistical inference was performed with within-subject permutation procedures to avoid pseudo-replication from treating multiple bouts from the same subject as independent observations.

## Data Availability

The datasets presented in this study can be found in online repositories. The names of the repository/repositories and accession number(s) can be found below: Human sleep recording data obtained from (1) the Sleep-EDF Database Expanded on Physionet (https://www.physionet.org/content/sleep-edfx/1.0.0/), (2) Mignot Nature Communications dataset supported by the National Sleep Research Resource (https://sleepdata.org/datasets/mnc), and (3) Bitbrain Open Access Sleep (BOAS) dataset (https://www.bitbrain.com/science/eeg-datasets). Mouse sleep recording data available at https://zenodo.org/records/5817119#.YdvvNC_kGTc and https://zenodo.org/records/5820559#.Ydvvcy_kGTc.
