# Supplementary material for: A data-driven measure of REM sleep propensity for human and rodent sleep
Source: Front Neurosci. 2026 Jun 9;20:1844209. doi: 10.3389/fnins.2026.1844209 (PMC13286888; doi:10.3389/fnins.2026.1844209)
Supplement: Supplementary file 1 [file Data_Sheet_1.pdf]

Supplementary Material

S1 MOUSE AND RAT (DARK PHASE) RESULTS

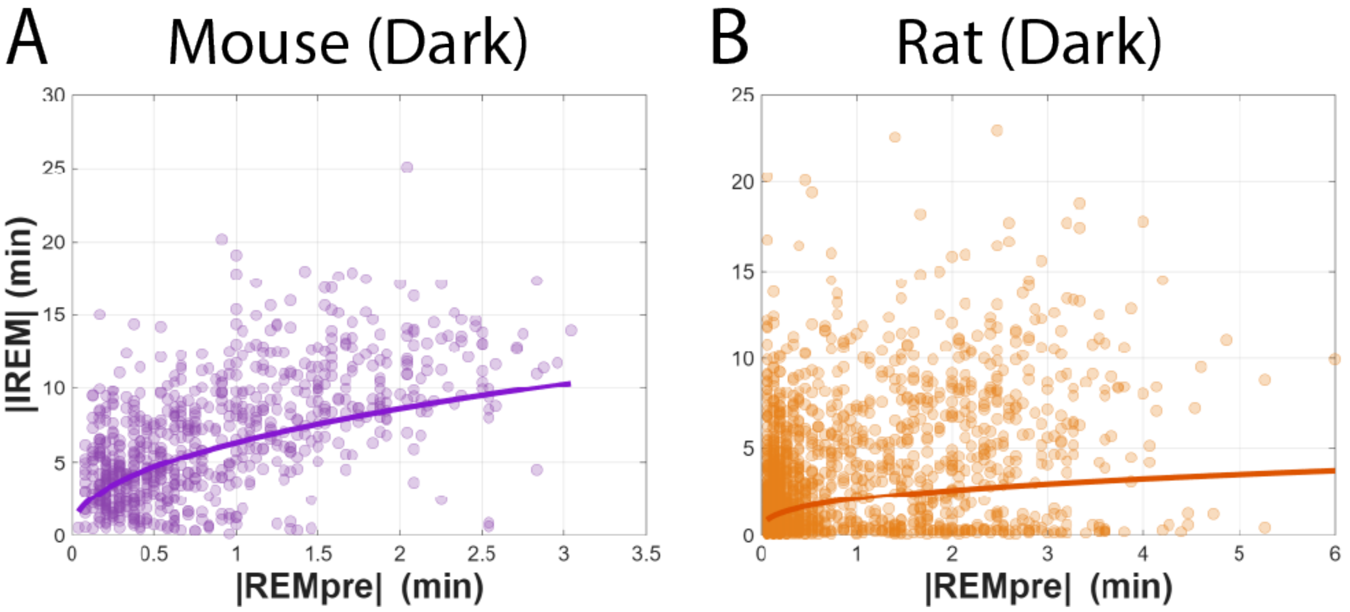

Figure S1. Inter-REMS interval duration $|\mathrm{IREM}|$ versus preceding REMS bout duration $|\mathrm{REMpre}|$ in mice and rats (dark phase). Scatter plots show the relationship between the duration of the preceding REMS bout ($|\mathrm{REMpre}|$) and the length of the subsequent inter-REMS interval ($|\mathrm{IREM}|$) for (A) mouse and (B) rat data in the dark phase. Each point represents a single REMS cycle. Solid lines show population-level fitted trends from log-log linear mixed-effects models with animal as a random intercept. In both species, the association between $|\mathrm{REMpre}|$ and $|\mathrm{IREM}|$ remained significantly positive after accounting for repeated REMS cycles contributed by the same animal: mouse dark, $\beta = 0.434$, $p = 3.95 \times 10^{-41}$; rat dark, $\beta = 0.318$, $p = 6.14 \times 10^{-35}$. Gamma-log generalized linear mixed-effects models and animal-level aggregated Spearman analyses gave consistent results in both datasets.

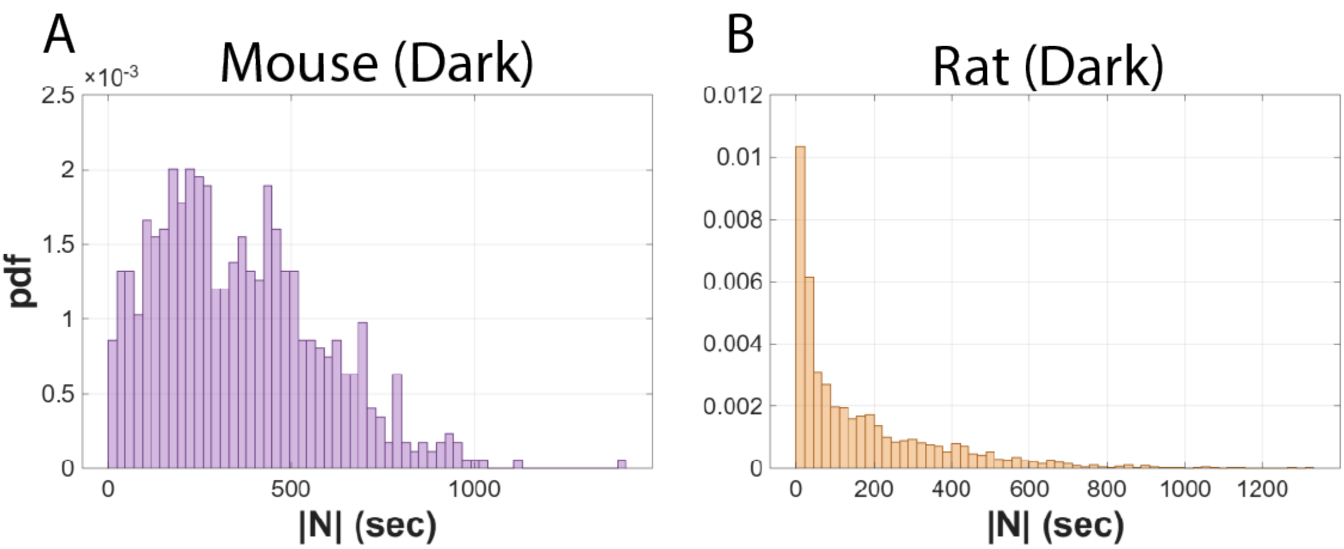

Figure S2. Empirical cumulative inter-REMS NREMS duration $|N|$ distributions in mice and rats (dark phase). Histograms of $|N|$ values pooled across all REMS cycles for (A) mouse and (B) rat data in the dark phase.

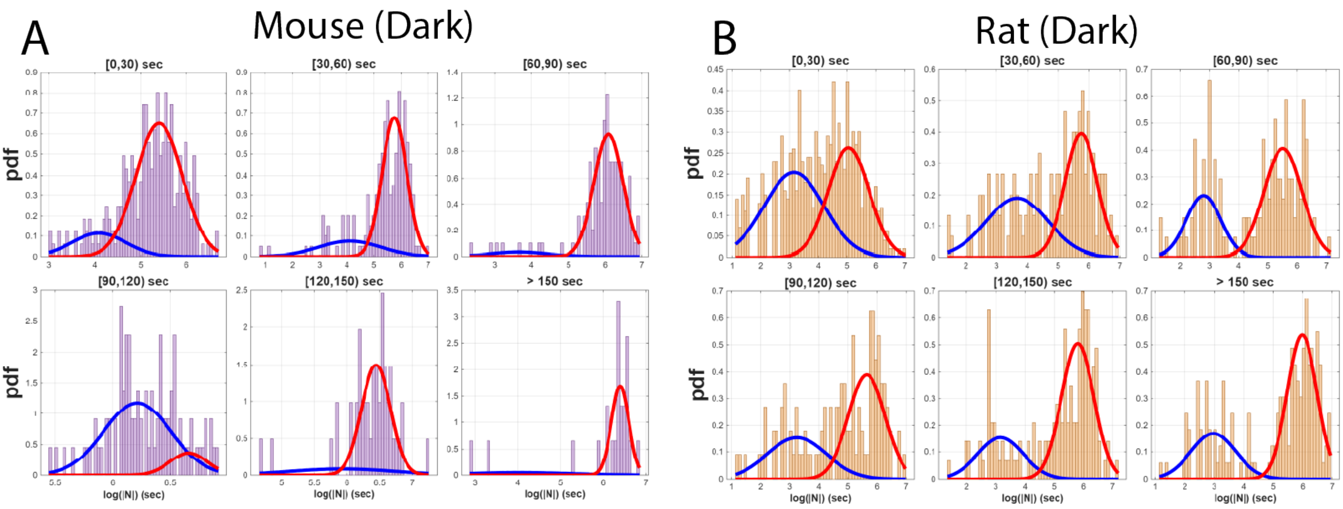

Figure S3. Gaussian mixture modeling (GMM) fits of the inter-REMS NREMS durations $|N|$ binned by $|\mathrm{REMpre}|$ duration for rodent data during the dark phase. (A) mouse (dark) and (B) rat (dark). Each panel shows the distribution of $\log(|N|)$ within a specific $|\mathrm{REMpre}|$ bin (labeled above each subplot). Within every bin, a two-component GMM is fit to the $\log(|N|)$ durations: the short-interval (sequential) mode (blue curve) and the long-interval (single) mode (red curve).

Table S1. Two-component Gaussian mixture model (GMM) parameters for mouse light-phase data after long-wake filtering ($\geq$ 2 min). The model was fit to $z = \log(|N|)$, where $|N|$ is NREMS duration in seconds. For each REMpre bin, n is the number of REMS cycles included, w_j is the mixing weight, μ_j is the mean, and σ_j is the standard deviation of component j ($j = 1, 2$) on the log scale. Components are ordered by increasing mean.

Bin	REMpre range (s)	n	w_1	μ_1	σ_1	w_2	μ_2	σ_2
1	$[0, 30)$	1764	0.570364	4.679818	0.747388	0.429636	5.584552	0.428502
2	$[30, 60)$	955	0.274363	4.139118	0.795511	0.725637	5.699581	0.457006
3	$[60, 90)$	538	0.211115	3.962436	1.006828	0.788885	6.073470	0.374800
4	$[90, 120)$	359	0.081130	3.715733	0.997149	0.918870	6.241783	0.335687
5	$[120, 150)$	214	0.060897	3.497884	0.701369	0.939103	6.341828	0.307352
6	$[150, \infty)$	175	0.060199	5.989144	0.585698	0.939801	6.501553	0.254413

Table S2. Two-component Gaussian mixture model (GMM) parameters for mouse dark-phase data after long-wake filtering ($\geq$ 2 min). The model was fit to $z = \log(|N|)$, where $|N|$ is NREMS duration in seconds. For each REMpre bin, n is the number of REMS cycles included, w_j is the mixing weight, μ_j is the mean, and σ_j is the standard deviation of component j ($j = 1, 2$) on the log scale. Components are ordered by increasing mean.

Bin	REMpre range (s)	n	w_1	μ_1	σ_1	w_2	μ_2	σ_2
1	$[0, 30)$	263	0.175742	4.118561	0.609007	0.824258	5.405572	0.508734
2	$[30, 60)$	190	0.222775	4.107440	1.201124	0.777225	5.755275	0.459099
3	$[60, 90)$	126	0.072323	3.638997	0.809157	0.927677	6.093499	0.397896
4	$[90, 120)$	90	0.854246	6.212131	0.291010	0.145754	6.656193	0.159850
5	$[120, 150)$	47	0.168139	5.926227	0.816934	0.831861	6.444326	0.221955
6	$[150, \infty)$	23	0.142958	4.037990	1.207258	0.857042	6.400520	0.202394

Table S3. Two-component Gaussian mixture model (GMM) parameters for rat light-phase data after long-wake filtering ($\geq$ 2 min). The model was fit to $z = \log(|N|)$, where $|N|$ is NREMS duration in seconds. For each REMpre bin, n is the number of REMS cycles included, w_j is the mixing weight, μ_j is the mean, and σ_j is the standard deviation of component j ($j = 1, 2$) on the log scale. Components are ordered by increasing mean.

Bin	REMpre range (s)	n	w_1	μ_1	σ_1	w_2	μ_2	σ_2
1	$[0, 30)$	1218	0.564796	3.130091	1.064274	0.435204	5.091941	0.687526
2	$[30, 60)$	217	0.504750	3.770096	1.129371	0.495250	5.716675	0.526266
3	$[60, 90)$	152	0.357612	2.829082	0.682314	0.642388	5.501217	0.693970
4	$[90, 120)$	129	0.545531	3.692626	1.203597	0.454469	5.878608	0.426530
5	$[120, 150)$	171	0.348783	3.318593	0.925519	0.651217	5.866989	0.504289
6	$[150, \infty)$	202	0.325012	2.920484	0.809527	0.674988	6.014627	0.493327

Table S4. Two-component Gaussian mixture model (GMM) parameters for rat dark-phase data after long-wake filtering ($\geq$ 2 min). The model was fit to $z = \log(|N|)$, where $|N|$ is NREMS duration in seconds. For each REMpre bin, n is the number of REMS cycles included, w_j is the mixing weight, μ_j is the mean, and σ_j is the standard deviation of component j ($j = 1, 2$) on the log scale. Components are ordered by increasing mean.

Bin	REMpre range (s)	n	w_1	μ_1	σ_1	w_2	μ_2	σ_2
1	$[0, 30)$	1012	0.536597	3.163676	1.059356	0.463403	5.046773	0.712614
2	$[30, 60)$	162	0.481607	3.678129	1.007557	0.518393	5.755845	0.524027
3	$[60, 90)$	140	0.338321	2.814225	0.554891	0.661679	5.515701	0.651625
4	$[90, 120)$	115	0.373850	3.174141	0.984049	0.626150	5.615120	0.638786
5	$[120, 150)$	147	0.301097	3.174769	0.789621	0.698903	5.814754	0.550853
6	$[150, \infty)$	169	0.321076	2.974512	0.744634	0.678924	5.986087	0.504694

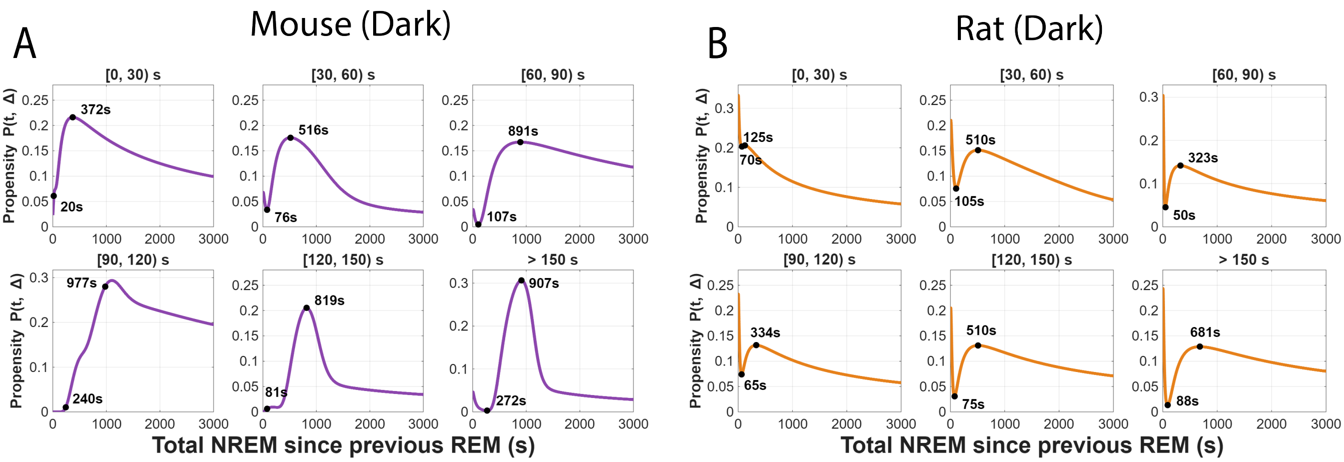

Figure S4. REMS propensity across $|\mathrm{REMpre}|$ bins for (A) mouse and (B) rat in the dark phase. For each species, the propensity function $P(t, \Delta)$ is computed within $|\mathrm{REMpre}|$-stratified bins.

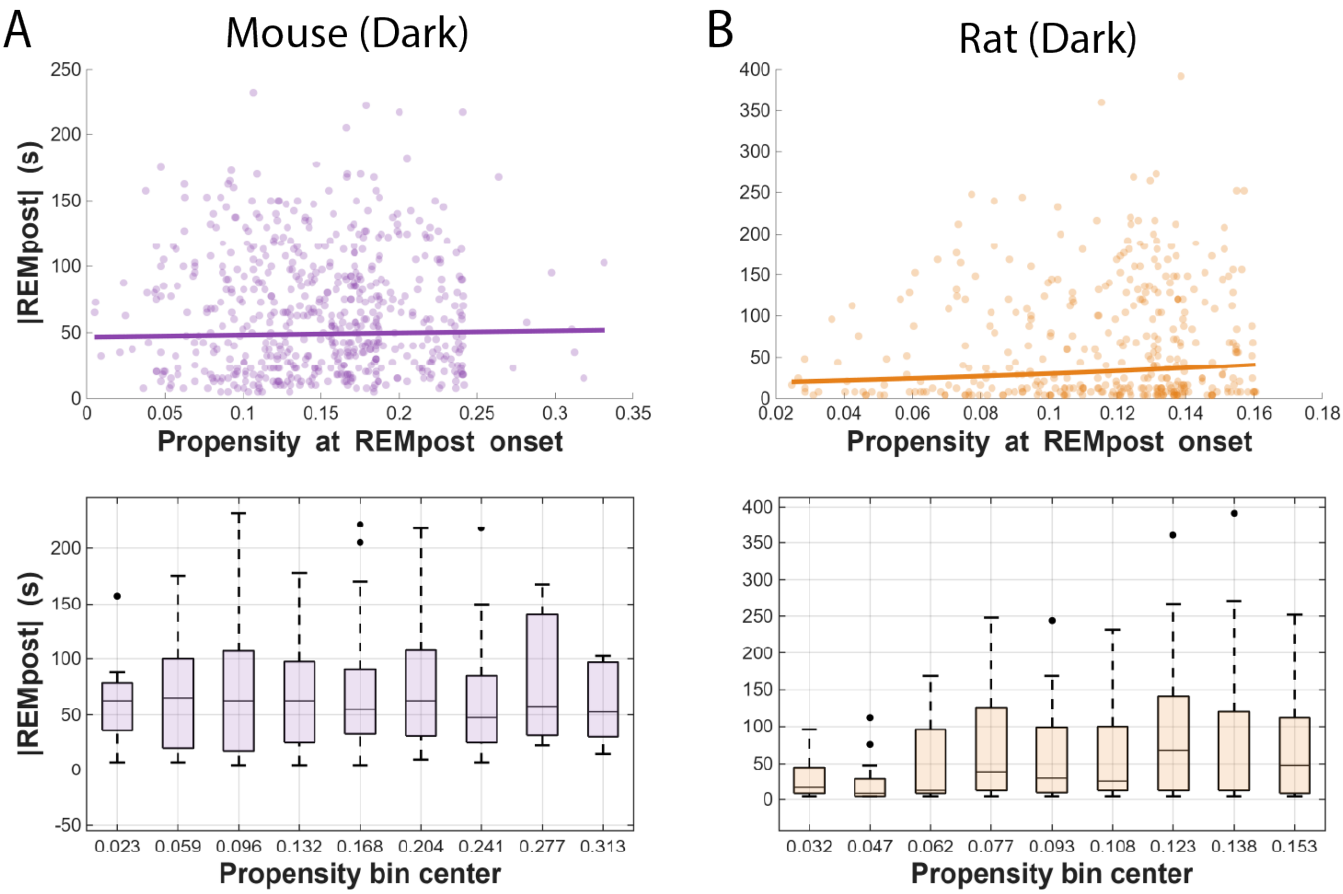

Figure S5. Top: Correlation between REMS propensity and the duration of the subsequent REMS bout $|\mathrm{REMpost}|$ for mice and rats dark-phase. Scatter plots show the relationship between the REMS propensity at REMS onset and the duration of the following REMS episode ($|\mathrm{REMpost}|$, in seconds) for mouse dark-phase and rat dark-phase REMS cycles. Each point represents a single REMS cycle, and solid lines show population-level fitted trends from mixed-effects models computed over cycles within the increasing-propensity regime. The estimated fixed-effect coefficient for propensity was not significant for mouse (dark) ($\beta = 0.314$, $p = 0.618$) but was positive and significant for rat (dark) ($\beta = 5.401$, $p = 0.0156$). Bottom: REMpost duration vs. REMS propensity at onset. Box-and-whisker summaries of $|\mathrm{REMpost}|$ (s) across propensity bins.

S2 DEFINITION AND FILTERING OF LONG-WAKE EPISODES

During sleep recordings, rodents and human periodically exhibit spontaneous wake periods between consecutive NREMS–REMS cycles. These wake bouts vary in duration, and exceptionally long episodes can perturb the temporal rhythm of the sleep cycle. To quantify their effect, we introduced a **Long-Wake (LW) filtering procedure** in which cycles containing a contiguous wake bout greater than a given threshold were excluded from subsequent analyses. The goal of this filtering step is to identify which threshold yields the most stable, well-structured distribution of NREMS durations, *i.e.*, which "Long-Wake" cutoff best captures the intrinsic rhythm of sleep without distortions from abnormally long awakenings. Specifically, thresholds of $\tau_{\text{thr}} = \{2,\ 5,\ 7,\ 10\}$ minutes were applied to human and both mouse and rat datasets under light and dark conditions. For each case, cycles satisfying "max contiguous wake duration $\geq \tau_{\text{thr}}$", were discarded, and all remaining cycles were retained for Gaussian Mixture Model (GMM) fitting of NREMS durations.

S2.1 Assessing Goodness of Fit: KS Test vs Model Selection Criteria

To evaluate how well the Gaussian Mixture Model (GMM) describes the observed NREMS duration data, one commonly used nonparametric measure is the Kolmogorov-Smirnov (KS) statistic. The KS statistic quantifies the largest vertical distance between the empirical cumulative distribution function (ECDF) of the data and the theoretical cumulative distribution function (CDF) predicted by the fitted model:

$$D_{\mathrm{KS}} = \sup_x \left| F_{\text{emp}}(x) - F_{\text{model}}(x) \right|,$$

where $F_{\text{emp}}(x)$ is the empirical CDF of the sample and $F_{\text{model}}(x)$ is the CDF derived from the GMM. We use the KS statistic to carry out a KS test corrected for the GMM, similarly to how the Lilliefors-corrected KS test corrects for a Gaussian distribution. In the corrected KS-test we use Monte-Carlo simulations to compute the probability distribution of

$$\tilde{D}_{\mathrm{KS}} = \sup_x \left| F_{\text{test}}(x) - F_{\text{model}}(x) \right|,$$

where $F_{\text{test}}(x)$ is an empirical CDF produced from n measurements of a random variable with CDF given by $F_{\text{model}}(x)$, n being equal to the number of samples used to compute $F_{\text{emp}}(x)$. If

$$\text{Probability}\left(\tilde{D}_{\mathrm{KS}} > D_{\mathrm{KS}}\right) < 0.05,$$

then the corrected KS-test rejects the null hypothesis that the fitted model is the distribution that produced the empirical CDF. In this context,

$$\text{p-value} = \text{Probability}\left(\tilde{D}_{\mathrm{KS}} > D_{\mathrm{KS}}\right).$$

Intuitively, the larger the D_{KS}, the worse the fit, and the smaller the p-value. Because smaller p-values for the corrected KS test indicate a worse fit, their interpretation differs from most goodness-of-fit tests, where lower values are desired.

Although the KS statistic is conceptually simple and distribution free, it is mainly sensitive to differences in the *central region* of the CDF, while being less responsive to discrepancies in the tails. Furthermore, when two parametric models are both close approximations to the same empirical distribution (as in our

GMMs under different Long-Wake thresholds), their D_{KS} values differ only marginally, often within the range of sampling noise (See tables S5- S11). Therefore, to obtain a more discriminative criterion that accounts for both fit accuracy and model complexity, we also used penalized-likelihood measures such as the “Bayesian Information Criterion (BIC)”, which better reflect the trade-off between descriptive power and parsimony in parametric models.

The KS-best distances in Table S5 quantify the maximum discrepancy between the empirical CDF of $z = \log(|N|)$ (log-NREMS duration) and the fitted two-component GMM CDF, after selecting the Expectation-Maximization (EM) algorithm restart that minimizes this discrepancy. Across datasets, the lumped fits have small KS distances ($D \approx 0.0187$–0.0449), indicating that a 2-component GMM provides a close overall approximation to the distribution of log-NREMS durations under the 2-min long-wake filtering rule. Within REMpre bins, most KS distances remain modest (typically $D \lesssim 0.06$), suggesting that the mixture model captures bin-specific distributions reasonably well. The largest discrepancies occur in later REMpre bins for darker-phase mouse data (Bin 6: $D = 0.0908$) and in Rat (Dark) Bin 4 ($D = 0.0884$), consistent with reduced sample sizes and/or more heterogeneous dynamics in the tail bins; nevertheless, these values still represent at most a $\sim$9% maximum CDF deviation. Overall, the table supports the conclusion that the fitted 2-GMMs track the empirical distributions of $\log(|N|)$ well across species/phase conditions, with the primary departures concentrated in a small number of tail bins.

Table S5. Kolmogorov–Smirnov (KS) distances D between empirical distributions of $z = \log(|N|)$ and fitted two–component Gaussian mixture models (2–GMM). Long-wake filtering: $\geq$ 2 min. Columns are species/phase; rows are REM$_{pre}$ bins (plus lumped).

	Mouse (Light)	Mouse (Dark)	Rat (Light)	Rat (Dark)
Lumped D	0.018669	0.044872	0.021688	0.020322
Bin 1 D	0.012145	0.022943	0.032828	0.028894
Bin 2 D	0.029810	0.056980	0.022256	0.028700
Bin 3 D	0.037001	0.035763	0.035950	0.037927
Bin 4 D	0.027220	0.053244	0.053032	0.088368
Bin 5 D	0.030804	0.067448	0.056791	0.069921
Bin 6 D	0.047724	0.090825	0.040919	0.038563

All four rodent datasets show KS diagnostic p-values exceeding 0.05 for the lumped fits and for every REMpre bin (Table S6), indicating no bin exhibits an obvious distributional mismatch between the empirical CDF of $z = \log(|N|)$ and the fitted two-component GMM. At the pooled (lumped) level, the p-values are moderate (Mouse Light: 0.11–0.12; Mouse Dark: ≈ 0.10; Rat Light: 0.28; Rat Dark: 0.46). Within bins, most p-values are large (often > 0.3 and frequently near 1), suggesting the fitted mixture CDF tracks the empirical CDF closely; the smallest bin-level p-values occur in Rat Light bin 1 ($p = 0.142$) and Rat Dark bin 4 ($p = 0.316$), but remain above the 0.05 threshold. Overall, these KS diagnostics support the adequacy of the two-component GMM approximation to log-NREMS durations across REMpre bins under the 2-min long-wake filtering rule (noting that KS p-values are used here as descriptive goodness-of-fit checks when model parameters are estimated from the data).

S2.2 Bayesian Information Criterion (BIC) Formulation

For each dataset, both a lumped model (one global 2-component GMM) and a per–bin model (2-component GMM fitted separately within each REMS preceding bin) were evaluated.

The Bayesian Information Criterion (BIC) is defined as

$$\mathrm{BIC} = -2\ln(\hat{L}) + p\ln(N),$$

Table S6. Kolmogorov–Smirnov (KS) p-values for two-component GMM fits to $z = \log(|N|)$ (NREMS duration in seconds), after long-wake filtering (≥ 2 min). Columns are species/phase; rows are REMpre bins (plus lumped).

	Mouse (Light)	Mouse (Dark)	Rat (Light)	Rat (Dark)
Lumped p	0.1213	0.0993	0.2766	0.4632
Bin 1 p	0.9560	0.9990	0.1421	0.3621
Bin 2 p	0.3590	0.5557	0.9999	0.9992
Bin 3 p	0.4458	0.9965	0.9881	0.9862
Bin 4 p	0.9503	0.9557	0.8519	0.3164
Bin 5 p	0.9859	0.9793	0.6273	0.4551
Bin 6 p	0.8112	0.9879	0.8813	0.9598

where $\hat{L}$ is the maximized likelihood of the fitted model, p is the number of free parameters, and N is the number of data points (that is, the number of cycles used in fitting). Lower BIC values indicate a better trade-off between goodness of fit and model complexity.

For a one-dimensional Gaussian mixture model with $K = 2$ components, the number of free parameters is

$$p = (K - 1) + 2K.$$

Here, $(K - 1)$ counts the independent mixing proportions, since the K component weights must sum to 1, leaving only $K - 1$ free. The term $2K$ counts the Gaussian parameters: each of the K components contributes one mean and one variance. Thus, for a two-component model,

$$p = (2 - 1) + 2(2) = 1 + 4 = 5.$$

For the per-bin model with six REM_{pre} bins, a separate two-component GMM is fit in each bin, so the total number of free parameters is

$$p_{\text{per-bin}} = 6 \times 5 = 30.$$

S2.3 BIC Results Across Long-Wake Thresholds

Tables S7- S11 summarize the BIC and KS results for mouse and rat datasets under Light and Dark conditions and human (note LL indicates Likelihood, BIC is Bayesian Information Criterion, and KS is Kolmogrov-Smirnov test).

Table S7. Mouse (Light) dataset under different long–Wake thresholds.

Thr (min)	Model	N	LL	BIC	KS
2	Lumped	4497	-5582.6	11207.0	0.0259
2	Per-bin	4497	-4400.0	**9052.4**	0.0266
5	Lumped	4673	-5824.3	11691.0	0.0250
5	Per-bin	4673	-4638.7	9530.8	0.0268
7	Lumped	4744	-5934.0	11910.0	0.0242
7	Per-bin	4744	-4757.8	9769.5	0.0261
10	Lumped	4836	-6076.5	12195.0	0.0238
10	Per-bin	4836	-4873.4	10001.0	0.0263

Across all datasets, the **2-minute Long–Wake threshold consistently yields the lowest BIC**, while KS differences remain negligible. This result shows that shorter Long–Wake filtering (≥ 2 min) best isolates the stable NREMS–REMS rhythm and removes irregular cycles that degrade the overall mixture structure.

Table S8. Mouse (Dark) dataset under different long–Wake thresholds.

Thr (min)	Model	N	LL	BIC	KS
2	Lumped	884	-981.9	1997.7	0.0306
2	Per-bin	884	-739.2	**1681.9**	0.0443
5	Lumped	923	-1013.2	2060.6	0.0282
5	Per-bin	923	-778.5	1761.9	0.0440
7	Lumped	943	-1033.3	2100.9	0.0276
7	Per-bin	943	-807.0	1819.4	0.0435
10	Lumped	969	-1057.7	2149.9	0.0271
10	Per-bin	969	-830.9	1868.1	0.0455

Table S9. Rat (Light) dataset under different long–Wake thresholds.

Thr (min)	Model	N	LL	BIC	KS
2	Lumped	2297	-3876.4	7791.4	0.0224
2	Per-bin	2297	-3637.4	**7506.9**	0.0394
5	Lumped	2448	-4132.6	8304.2	0.0213
5	Per-bin	2448	-3869.3	7972.7	0.0384
7	Lumped	2481	-4185.4	8409.8	0.0214
7	Per-bin	2481	-3918.4	8071.4	0.0372
10	Lumped	2520	-4237.1	8513.4	0.0219
10	Per-bin	2520	-3972.1	8179.2	0.0372

Table S10. Rat (Dark) dataset under different long–Wake thresholds.

Thr (min)	Model	N	LL	BIC	KS
2	Lumped	1918	-3203.5	6444.8	0.0261
2	Per-bin	1918	-2991.4	**6209.7**	0.0400
5	Lumped	2034	-3401.2	6840.4	0.0252
5	Per-bin	2034	-3164.0	6556.6	0.0410
7	Lumped	2062	-3445.2	6928.5	0.0266
7	Per-bin	2062	-3203.1	6635.1	0.0393
10	Lumped	2098	-3492.8	7023.8	0.0273
10	Per-bin	2098	-3236.7	6702.8	0.0409

Table S11. Human dataset under different long–Wake thresholds.

Thr (min)	Model	N	LL	BIC	KS
2	Lumped	3397	-5644.8	11330	0.1134
2	Per-bin	3397	-5529.2	**11262**	0.1062
5	Lumped	3786	-6360.5	12762	0.1021
5	Per-bin	3786	-6256.1	12718	0.0943
7	Lumped	3877	-6506.9	13055	0.1010
7	Per-bin	3877	-6413.4	13033	0.0970
10	Lumped	3958	-6650.7	13343	0.0985
10	Per-bin	3958	-6556.5	13320	0.0949

The comparative analyses across all rodent datasets show that simple KS-based tests are insufficient for discriminating subtle changes in model performance under different Long–Wake thresholds. In contrast, the Bayesian Information Criterion (BIC) provides a more sensitive and integrative measure by penalizing overfitting while rewarding explanatory accuracy. The BIC consistently identified the 2-minute Long–Wake threshold as yielding the best model fit for both Mouse and Rat, under both light and dark phases. This suggests that filtering out wake bouts longer than approximately 2 minutes produces a more homogeneous and physiologically coherent dataset of NREMS cycles. From a biological perspective, these findings have

implications for understanding the homeostatic regulation of NREM sleep. Long continuous wake episodes appear to disrupt the stability and timing of subsequent NREMS periods, leading to greater variance and irregularity in cycle duration. This increase in variability, confirmed through variance-ratio analysis and probability density histograms, indicates that NREMS homeostasis becomes temporarily dysregulated after extended Wake. Such episodes may reflect an overshoot or delayed recovery in the sleep–wake control system, possibly linked to altered neuronal excitability or reduced synchronization in cortical slow-wave activity.

Therefore, the 2-minute cutoff represents not only a statistical optimum but also a physiologically meaningful boundary between short wake bouts and disruptive wake episodes. By excluding these prolonged wake episodes, the resulting data more faithfully capture the intrinsic rhythm of sleep–wake alternation, allowing for more accurate modeling of NREMS homeostatic dynamics and IREM stability.

S3 HUMAN MODEL COMPARISON, SELECTION, AND PROPENSITY ANALYSIS

S3.1. Candidate model families for inter–REMS NREMS duration

Let $T = |N|$ denote the human inter–REMS NREMS duration (minutes). The empirical distribution of T exhibits strong right skewness, heavy tails, and a pronounced spike at the measurement floor $x_{\min} = 0.5$ minutes. To determine an appropriate statistical model, we considered several increasingly flexible parametric families.

S3.1.1. Two-component continuous mixtures.

As an initial approach, we considered standard two-component mixtures defined on $(0, \infty)$:

(a) Exponential + Lognormal (EXP+LN),
(b) Weibull + Lognormal (WEI+LN),
(c) Lognormal + Lognormal (LN+LN).

These models assume

$$f(t) = w f_S(t) + (1 - w) f_L(t), \qquad 0 < w < 1,$$

where f_S represents a short-duration component and f_L a long-duration component. The exponential model imposes memoryless short-bout dynamics. The Weibull allows flexible short-bout hazard shapes. The lognormal components allow heavy-tailed behavior.

S3.1.2. Three-component mixtures.

To capture possible intermediate-duration structure, we also evaluated three-component families such as:

$$\text{WEI+LN+LN}, \quad \text{LN+LN+LN}, \quad \text{WEI+LN+LNP},$$

where LNP denotes a lognormal–Pareto heavy-tail extension.

S3.1.3. Models with an explicit atom at $x_{\min}$.

Inspection of the empirical distribution revealed a substantial spike at $x_{\min} = 0.5$ minutes. When purely continuous mixtures were fit to these data, the short-duration component was forced to account for the excess probability mass at the measurement floor. This often led to sensitive or inconsistent estimates of

the short-component parameters across candidate models and fitting runs, and could also degrade the fit to the remaining continuous part of the distribution, including the upper tail. To separate the point mass at the measurement floor from the continuous behavior for $T > x_{\min}$, we therefore considered models with an explicit *atom* at $x_{\min}$; that is, a point mass is assigned to the event $T = x_{\min}$:

$$\Pr(T = x_{\min}) = a, \qquad \Pr(T > x_{\min}) = 1 - a,$$

while the remaining probability mass is modeled by a continuous mixture on $[x_{\min}, \infty)$.

S3.2. Model selection criteria

Selecting an appropriate parametric model for human inter–REMS NREMS durations requires balancing three distinct objectives: *(i)* accurate representation of the empirical distribution, *(ii)* parsimony and interpretability, and *(iii)* statistical validity of goodness-of-fit inference under parameter estimation. No single metric captures all three simultaneously. For this reason, we adopted a layered evaluation framework combining information-theoretic criteria and distributional diagnostics.

First, we use likelihood-based criteria to compare models relative to one another; these likelihood-based measures are described in Section S2.1 below. These criteria evaluate how well a model explains the observed data while penalizing unnecessary complexity. However, good relative performance does not guarantee that a model provides an adequate absolute description of the data.

Second, we compute global distributional discrepancy measures that compare the fitted cumulative distribution function (CDF) directly against the empirical CDF, as described in S2.2 below. These metrics assess whether systematic deviations remain, particularly in the tails or near structural boundaries such as $x_{\min}$.

Third, because parameters are estimated from the same data used to assess fit, classical goodness-of-fit p-values are not valid. We therefore employ a refit parametric bootstrap, described in S2.3 below, to properly calibrate the null distribution of the test statistic.

The specific tools used in this layered framework are described below.

S3.2.1. Likelihood and BIC (relative fit).

For each candidate model, we computed the maximized log-likelihood $\ell(\hat{\theta})$ and the Bayesian Information Criterion

$$\mathrm{BIC} = -2\ell(\hat{\theta}) + k \log(n),$$

where k is the number of free parameters. BIC penalizes additional parameters and favors parsimonious structure.

Across the families considered, models incorporating an explicit atom at $x_{\min}$ consistently improved the likelihood relative to purely continuous mixtures, reflecting the substantial empirical mass at the measurement floor. Among the atom-based candidates, the two-component continuous structure consisting of an E1-short component (a short-duration density proportional to $\exp(-rt)/t$ on $[x_{\min}, \infty)$) and a truncated normal (TN) long-duration component achieved lower BIC values than more flexible three-component alternatives once the complexity penalty was taken into account. This indicates that the Atom + E1-short + truncated-normal formulation provides the most parsimonious representation of the data among the interpretable candidate families considered. Accordingly, subsequent analyses of goodness-of-fit diagnostics and propensity estimation were based on this selected model.

S3.2.2. Kolmogorov–Smirnov distance (absolute fit).

To assess absolute agreement between the fitted model and the empirical distribution, we computed the Kolmogorov–Smirnov (KS) distance

$$D_{\text{obs}} = \sup_t \left|F_n(t) - F_{\hat{\theta}}(t)\right|,$$

where n is the number of observations, F_n is the empirical CDF, and $F_{\hat{\theta}}$ is the fitted model CDF (including the atom at x_{min}). The KS distance summarizes the largest vertical deviation between the two CDFs and is sensitive to mismatches anywhere on the support, including near the boundary and in the tail. We use D_{obs} as a global diagnostic of distributional adequacy for the pooled dataset and for each REM–pre stratum.

The KS distance summarizes the largest vertical deviation between the two CDFs, and is sensitive to mismatches anywhere on the support, including near the boundary and in the tail. We use D_{obs} as a global diagnostic of distributional adequacy for the pooled dataset and for each REM–pre stratum.

S3.2.3. Refit parametric bootstrap (calibrated p-values).

When parameters are estimated from the same data used to compute the KS statistic, the classical KS null distribution does not apply (Stute et al., 1993). To obtain calibrated p-values, we used a refit parametric bootstrap:

1. Simulate a synthetic dataset of size n from the fitted model $F_{\hat{\theta}}$ (including the atom at x_{min}),
2. Refit the *same* model to the synthetic data using the identical estimation procedure (multi-start expectation–maximization (EM) with the same constraints), obtaining $\hat{\theta}^{(b)}$. Let $F_n^{(b)}$ denote the empirical CDF of the bth synthetic sample.
3. Compute the bootstrap KS statistic

$$D^{(b)} = \sup_t \left|F_n^{(b)}(t) - F_{\hat{\theta}^{(b)}}(t)\right|,$$

4. Repeat steps 1–3 independently for $b = 1, \ldots, B$, and estimate

$$p = \frac{1 + \sum_{b=1}^{B} \mathbf{1}\{D^{(b)} \geq D_{\text{obs}}\}}{B+1}.$$

This procedure properly accounts for parameter-estimation variability because the model is re-estimated for each synthetic dataset before the KS statistic is recomputed. Consequently, the bootstrap null distribution reflects both sampling variability and the additional uncertainty introduced by parameter fitting. Goodness-of-fit results (including D_{obs} and refit-bootstrap p-values for the pooled sample and each REM–pre bin) are reported in S12.

S3.3. Why the Atom + E1 + Truncated-Normal model was selected

Because the data are only observed for $t \geq x_{\text{min}}$, there is no empirical information about behavior below the measurement floor. In particular, although the empirical CDF has a positive jump at x_{min}, any hypothetical continuous behavior for $t < x_{\text{min}}$ is not identifiable from the data and therefore should not be treated as part of the fitted continuous regime.

Although LN+LN and certain three-component mixtures achieved lower BIC than simple two-component EXP+LN or WEI+LN, the final Atom + E1 + TN model was selected based on two overarching considerations:

(i) **Boundary-aware, interpretable decomposition.** The empirical distribution exhibits a pronounced lower-bound spike at the measurement floor $x_{\min}$ together with an approximately $1/t$-like decay in the density over short durations. Purely continuous mixture models on $(0, \infty)$ do not accommodate this boundary structure well: a $1/t$-type behavior cannot extend to 0 in a normalizable continuous density, whereas the observed data are naturally bounded below by $x_{\min} = 0.5$ minutes. By truncating the continuous model to $[x_{\min}, \infty)$ and assigning an explicit atom at $x_{\min}$, the model separates measurement-floor effects from the continuous short-duration regime. Conditional on $T > x_{\min}$, the E1-short component captures the observed near-boundary decay, while the truncated-normal component provides an interpretable representation of sustained NREMS episodes.

(ii) **Stability for downstream propensity analysis with minimal complexity.** Propensity is highly sensitive to model behavior near $x_{\min}$. Separating the atom prevents artificial lower-bound spikes in propensity curves. At the same time, compared with more flexible three-component mixtures, the Atom + two continuous components model retains interpretability while avoiding unnecessary complexity that can reduce parameter stability and complicate propensity estimation.

Thus, the final model balances boundary fidelity, interpretability, and stable downstream propensity estimation.

S3.4. Final model specification

Atom at the measurement floor.

We explicitly model the large spike observed at $x_{\min}$ by assigning a point mass (atom) at the floor:

$$\Pr(T = x_{\min}) = a.$$

Here $a \in (0, 1)$ represents the fraction of cycles recorded exactly at the floor value. Treating this spike as a discrete component prevents the continuous densities from being distorted to accommodate an artifact of discretization/measurement resolution.

Continuous mixture on $[x_{\min}, \infty)$*.*

Conditional on exceeding the floor ($T > x_{\min}$), durations are modeled by a two-component continuous mixture:

$$f_c(t) = w f_{\mathrm{E1}}(t; r) + (1 - w) f_{\mathrm{TN}}(t; \mu, \sigma), \qquad t \geq x_{\min}.$$

The mixing weight $w \in (0, 1)$ captures the probability that a continuous duration belongs to the short-duration regime, while $1 - w$ corresponds to the long-duration regime.

Short component (E1-short).

The short-duration component is designed to capture the high density near $x_{\min}$ together with an initially slowly decaying tail. It is defined on $[x_{\min}, \infty)$ by a normalized $\exp(-rt)/t$ form. The normalization

constant uses the exponential integral function

$$E_1(z) \;=\; \int_z^\infty \frac{e^{-u}}{u}\,du, \qquad z > 0.$$

With this choice, the short-component PDF is

$$f_{\mathrm{E1}}(t;r) = \frac{e^{-rt}}{t\,E_1(rx_{\min})}, \qquad t \ge x_{\min},$$

which is properly normalized on $[x_{\min}, \infty)$:

$$\int_{x_{\min}}^\infty f_{\mathrm{E1}}(t;r)\,dt = 1.$$

This component behaves like $1/t$ near the lower bound (supporting a large near-$x_{\min}$ density) while retaining an exponential tail e^{-rt} that prevents unrealistically heavy long-duration mass.

Long component (truncated normal).

Long durations are modeled by a normal distribution with mean μ and standard deviation σ, truncated to the physically admissible range $[x_{\min}, \infty)$. Its pdf is

$$f_{\mathrm{TN}}(t;\mu,\sigma) = \frac{\phi((t-\mu)/\sigma)}{\sigma\Big(1 - \Phi((x_{\min}-\mu)/\sigma)\Big)}, \qquad t \ge x_{\min},$$

where ϕ and Φ are the standard normal pdf and CDF, respectively. Truncation ensures the model assigns no probability mass below the measurement floor, while (μ, σ) provide an interpretable summary of the center and spread of sustained NREMS episodes.

S3.5. Parameter estimation

The atom mass a is estimated directly as the empirical proportion of observations equal to $x_{\min}$. Continuous parameters (w, r, μ, σ) are estimated using multi-start Expectation–Maximization (EM) applied to the subset $\{x_i > x_{\min}\}$. Multiple random initializations are used to reduce sensitivity to local optima, and the solution with highest log-likelihood is retained.

Given current parameters, posterior responsibilities for the short component are computed as

$$\gamma_i = \frac{w f_{\mathrm{E1}}(x_i;r)}{w f_{\mathrm{E1}}(x_i;r) + (1-w) f_{\mathrm{TN}}(x_i;\mu,\sigma)}.$$

Parameter updates are obtained via weighted maximum likelihood. Because closed-form updates are not available for r, numerical optimization is performed in the M-step. To preserve identifiability and prevent degeneracy, bounds are imposed on r, σ, and μ, and mixture weights are constrained to remain strictly between 0 and 1. Convergence is declared when the relative change in log-likelihood falls below a predefined tolerance.

S3.6. Goodness-of-fit results

For each pooled and REM–pre stratified fit, we report the estimated atom mass $\hat{a}$ at the measurement floor $x_{\min}$, the continuous mixture weight $\hat{w}$ corresponding to the short-duration component, and the component parameters $(\hat{r}, \hat{\mu}, \hat{\sigma})$ governing the E1-short and truncated-normal long regimes. We additionally report the maximized log-likelihood and the Bayesian Information Criterion (BIC) for model comparison, together with the observed discrete Kolmogorov–Smirnov statistic and its calibrated refit-bootstrap p-value. These quantities jointly summarize relative model performance, absolute goodness-of-fit, and the structural decomposition of short- and long-duration NREMS dynamics across pooled and REM–pre stratified analyses.

Because the observed NREMS durations $|N|$ are recorded exactly on a 0.5-min grid, goodness-of-fit was assessed using the corresponding *discrete* model rather than by comparing the empirical sample directly to the latent continuous distribution. To account for possible ambiguity in how a latent continuous duration is represented on the observation grid, we evaluated three mapping rules: floor, ceil, and nearest. For the pooled human data, goodness-of-fit was computed under each of these mappings, and the rule used for the REM–pre stratified analyses was selected according to the largest refit-bootstrap KS p-value in the pooled analysis. Under this comparison, the *ceil* mapping yielded the strongest agreement with the empirical grid-valued distribution and was therefore used for the REM–pre stratified goodness-of-fit calculations.

For a fixed mapping rule, the fitted atom + E1-short + truncated-normal model induces a probability mass function on the 0.5-min grid, from which a model-based discrete cumulative distribution function is obtained. The reported KS statistic is the maximum absolute difference between the empirical grid-based cumulative distribution function and this fitted discrete cumulative distribution function. The associated p-value was obtained by refit parametric bootstrap: synthetic datasets of the same size were generated from the fitted model, mapped to the grid using the same observation rule, refit by the same estimation procedure, and then re-evaluated using the same discrete KS statistic. This calibration accounts for parameter-estimation uncertainty and for the discretized nature of the observed data.

For the pooled human data, the observed discrete KS statistic under the selected *ceil* mapping was $D_{\text{obs}} = 0.0168$, with refit-bootstrap $p = 0.20$, indicating that the fitted model provides an adequate absolute description of the grid-valued empirical distribution. The same conclusion held across REM–pre strata. Specifically, for Bin 1 ($0.5 \leq \text{REMpre} < 5$ min), $D_{\text{obs}} = 0.0237$ with $p = 0.215$; for Bin 2 ($5 \leq \text{REMpre} < 12$ min), $D_{\text{obs}} = 0.0194$ with $p = 0.500$; and for Bin 3 ($12 \leq \text{REMpre} < 71.5$ min), $D_{\text{obs}} = 0.0262$ with $p = 0.115$. Thus, the observed KS discrepancies were typical under the fitted model once refitting uncertainty was taken into account, and the Atom + E1-short + truncated-normal model was not rejected as an absolute distributional description of the human $|N|$ data.

Table S12. Goodness-of-fit summary for the Atom + E1-short + Truncated-Normal model. The reported KS statistic is the discrete Kolmogorov–Smirnov distance computed on the 0.5-min observation grid under the selected discretization rule (*ceil*). All p-values were obtained from refit parametric bootstrap.

**Fit**	$\hat{a}$	$\hat{w}_{\text{short}}$	$\hat{r}$	$\hat{\mu}$	$\hat{\sigma}$	BIC	D_{obs}	p
Lumped	0.2922	0.7481	0.1081	65.5819	16.6177	11046.05	0.0168	0.200
Bin 1 $[0.5, 5)$	0.3541	0.9519	0.0246	4.0000	1.6171	5656.38	0.0237	0.215
Bin 2 $[5, 12)$	0.3056	0.7733	0.1126	64.0311	16.4390	3140.80	0.0194	0.500
Bin 3 $[12, 71.5)$	0.1470	0.5231	0.0630	69.4299	16.2589	3812.39	0.0262	0.115

S3.7. Propensity estimation

We define discrete-time propensity over a window Δ as

$$p_\Delta(t) = \frac{F_c(t+\Delta) - F_c(t)}{1 - F_c(t)},$$

where F_c denotes the CDF of the continuous component only. Throughout, $\Delta = 0.5$ minutes (30 seconds).

Why the continuous CDF is used.

The full fitted CDF has the form

$$F(t) = \begin{cases} 0, & t < x_{\min}, \\ a + (1-a)F_c(t), & t \geq x_{\min}, \end{cases}$$

where a is the atom mass at $x_{\min}$. For the propensity analysis, however, we are interested in termination dynamics conditional on the episode having already exceeded the measurement floor. Accordingly, for times $t > x_{\min}$, the relevant distributional behavior is described by the continuous component F_c, not by the atom at $x_{\min}$. If the full CDF F were used directly in the propensity definition, then near $t = x_{\min}$ the numerator would contain the discrete jump a, producing

$$F(t+\Delta) - F(t) \approx a + \text{(continuous contribution)}.$$

Because the survival term in the denominator is also altered, this discrete jump can generate an artificial spike in

$$p_\Delta(t) = \frac{F(t+\Delta) - F(t)}{1 - F(t)},$$

immediately above $x_{\min}$. This spike does not represent genuine termination dynamics of continuous NREMS durations; rather, it reflects discretization and measurement-floor effects. Including the atom in the propensity calculation therefore conflates two distinct mechanisms: (1) the structural measurement-floor mass at $x_{\min}$, and (2) the intrinsic termination dynamics of continuous NREMS episodes.

To isolate the latter, we compute propensity using F_c, the CDF of the continuous mixture only. This ensures that propensity reflects

$$\Pr(T \in [t, t+\Delta) \mid T \geq t,\ T > x_{\min}),$$

i.e., termination dynamics conditional on having already exceeded the measurement floor. Note this modification affects only the propensity calculation, not the fitted model itself. Even when using F_c, numerical instability can arise exactly at $t = x_{\min}$ because of truncation. We therefore evaluate propensity beginning at

$$t_{\text{start}} = x_{\min} + 0.05,$$

where 0.05 minutes corresponds to 3 seconds. This small offset removes residual boundary artifacts without altering the substantive shape of the curve.

S4 HYPOTHESIS TESTS FOR NORMALIZED-TIME REMS DISTRIBUTIONS AND ONSET-DECILE REMS BOUT DURATION (FIGURES 7-9)

To complement the descriptive bar plots, we carried out subject-level permutation-based hypothesis tests designed to assess whether the observed decile structure reflects a reproducible pattern across subjects rather than only pooled event counts. Because these analyses are based on repeated measurements within subject, we avoided tests that would treat all REMS bouts as independent observations.

Edge-versus-middle enrichment of REMS onset fractions

Rather than fitting a linear trend across normalized-time deciles, we tested whether REMS onset fractions near the beginning and end of the sleep episode differed from the corresponding fractions in the middle of the sleep episode. For each subject i, let $x_{i,1}$ denote the fraction of that subject's nightly REMS onsets falling in the first decile (0–10% of normalized sleep time), let $x_{i,10}$ denote the fraction falling in the last decile (90–100%), and define the subject-specific middle-decile mean by

$$\bar{x}_{i,\text{mid}} = \frac{1}{8}\sum_{d=2}^{9} x_{i,d}.$$

We then formed three paired contrasts:

$$d_i^{(1)} = x_{i,1} - \bar{x}_{i,\text{mid}},$$

$$d_i^{(10)} = x_{i,10} - \bar{x}_{i,\text{mid}},$$

and

$$d_i^{(\text{edge})} = \frac{x_{i,1} + x_{i,10}}{2} - \bar{x}_{i,\text{mid}}.$$

For each contrast, the null hypothesis was that the mean paired difference across subjects was zero,

$$H_0 : \mathbb{E}[d_i] = 0,$$

against the two-sided alternative

$$H_A : \mathbb{E}[d_i] \neq 0.$$

To evaluate these hypotheses without imposing a Gaussian assumption on subject-level fractions, we used a two-sided paired sign-flip permutation test. For a given set of subject-level paired differences $d_1, \ldots, d_n$, the observed test statistic was the sample mean

$$T_{\text{obs}} = \frac{1}{n}\sum_{i=1}^{n} d_i.$$

Under the null hypothesis of no systematic edge-versus-middle effect, the sign of each paired difference is exchangeable. We therefore generated a null distribution by repeatedly multiplying each d_i by an independent random sign $s_i \in \{-1, +1\}$ with equal probability and recomputing

$$T^{(b)} = \frac{1}{n}\sum_{i=1}^{n} s_i d_i, \qquad b = 1, \ldots, B.$$

Using $B = 20{,}000$ random sign-flip resamples, the two-sided permutation p-value was estimated as

$$p = \frac{1}{B} \sum_{b=1}^{B} \mathbf{1}\Big(|T^{(b)}| \geq |T_{\text{obs}}|\Big) .$$

This procedure was applied separately to the first-decile contrast, the last-decile contrast, and the averaged-edge contrast.

These tests assess whether the subject-level REMS onset fraction at the beginning of the night, at the end of the night, or averaged across both the first and last decile differs from the corresponding subject-level average fraction across the middle 10–90% of normalized sleep time. Because the analysis is performed at the subject level, the inference reflects between-subject consistency of edge enrichment at the beginning and end of the sleep period rather than only pooled event counts.

Association between REMS bout duration and onset decile

We next tested whether REMS bout duration varies across normalized sleep-time onset deciles. Although the corresponding figure displays pooled bout-level means, direct inference at the bout level would treat multiple bouts from the same subject as independent observations. To avoid this pseudo-replication, we used a subject-level within-subject permutation framework.

For each subject i and onset decile d, we computed the mean REMS bout duration among that subject's bouts assigned to decile d:

$$m_{i,d} = \frac{1}{n_{i,d}} \sum_{c \in \mathcal{B}_{i,d}} Y_c,$$

where $\mathcal{B}_{i,d}$ denotes the set of bouts from subject i in decile d, $n_{i,d} = |\mathcal{B}_{i,d}|$, and Y_c is the REMS bout duration of bout c. If a subject had no bouts in a given decile, the corresponding entry was treated as missing. This produced a subject-by-decile matrix

$$M = (m_{i,d}),$$

with one row per subject and one column per onset decile.

We tested the global null hypothesis that REMS bout duration is unrelated to onset decile:

$$H_0 : \text{ within each subject, onset-decile labels carry no information about REMS bout duration,}$$

against the alternative

$$H_A : \text{ REMS bout duration depends on onset decile.}$$

Equivalently, under H_0, onset-decile labels are exchangeable within subject.

To quantify decile-dependent structure while controlling for subject-specific baseline differences in REMS bout duration, we first centered each subject's row by subtracting that subject's mean across observed deciles:

$$\tilde{m}_{i,d} = m_{i,d} - \bar{m}_i, \qquad \bar{m}_i = \frac{1}{|\mathcal{D}_i|} \sum_{d \in \mathcal{D}_i} m_{i,d},$$

where $\mathcal{D}_i$ is the set of onset deciles observed for subject i. We then computed the decile-specific centered mean across subjects,

$$\tilde{\mu}_d = \frac{1}{N_d} \sum_{i:\, d \in \mathcal{D}_i} \tilde{m}_{i,d},$$

where N_d is the number of subjects contributing data to decile d. The observed test statistic was the summed squared decile effect,

$$T_{\text{obs}} = \sum_{d=1}^{10} \tilde{\mu}_d^2.$$

Large values of T_{obs} indicate systematic variation in subject-centered REMS bout duration across onset deciles.

To generate the null distribution, we permuted onset-decile labels *within each subject*; that is, for each subject, we randomly reassigned that subject's observed decile labels across that subject's bouts while leaving the REMS bout durations unchanged. This preserves the number of bouts contributed by each subject, the subject-specific distribution of REMS bout durations, and the marginal number of observations per subject, while destroying any systematic association between REMS bout duration and onset decile within subject.

For each permutation $b = 1, \ldots, B$, we recomputed the subject-level decile means, the centered decile effects, and the corresponding test statistic $T^{(b)}$. Using $B = 20{,}000$ within-subject permutations, the permutation p-value was estimated as

$$p = \frac{1}{B} \sum_{b=1}^{B} \mathbf{1}\left(T^{(b)} \geq T_{\text{obs}}\right).$$

This test evaluates whether the decile at which a REMS bout begins is associated with REMS bout duration, after accounting for subject-specific baseline differences and without assuming a linear trend across the night. A significant result indicates that REMS bout duration varies across onset deciles, but does not by itself imply a monotone increase or decrease.

S5 STATISTICAL ANALYSES FOR FIGURES 2 AND 6

Linear mixed-effects models

For each figure, our primary analysis used a linear mixed-effects model (Pinheiro and Bates, 2000; Bates et al., 2015) with a random intercept for subject or animal. This structure accounts for repeated REMS cycles contributed by the same individual while still using all retained cycles in the analysis.

For Figure 2, which examines the association between preceding REMS bout duration and the subsequent inter-REMS interval, the main model for the human data was

$$\log(|\text{IREM}|_{ij}) = \beta_0 + \beta_1 \log(|\text{REMpre}|_{ij}) + \beta_2 \,\text{Source}_{ij} + u_i + \varepsilon_{ij},$$

where $|\text{IREM}|_{ij}$ denotes the inter-REMS interval for cycle j from subject i, $|\text{REMpre}|_{ij}$ is the duration of the REMS bout initiating that cycle, Source_{ij} is a categorical fixed effect indicating the source dataset for that observation, u_i is a subject-specific random intercept, and ε_{ij} is the residual error term. In the human

analysis, the source term was included to adjust for systematic baseline differences among the contributing human datasets (Sleep-EDF, MNC, and Bitbrain/BOAS), while estimating a common association between $|\mathrm{REMpre}|$ and $|\mathrm{IREM}|$ across datasets. The random intercept u_i allows each subject to have a subject-specific baseline level of the response, thereby accounting for within-subject dependence among multiple REMS cycles contributed by the same individual. In implementation, subject grouping was defined from the subject identifier available in the data table and model fitting used reference coding for categorical effects. For rodent data, analogous models were fit separately for each species and light/dark condition, with animal as the random intercept and without a source term, because each rodent dataset/condition was analyzed separately rather than pooled across distinct acquisition sources.

For Figure 6, which examines the association between REMS propensity at REMS onset and the duration of the subsequent REMS bout, the main model for the human data was

$$\log(|\mathrm{REMpost}|_{ij}) = \alpha_0 + \alpha_1 P_{ij} + \alpha_2\, \mathrm{Source}_{ij} + u_i + \varepsilon_{ij},$$

where P_{ij} is the REMS propensity value evaluated at REMS onset for cycle j from subject i. As above, Source_{ij} adjusts for baseline differences among human datasets, while u_i represents a subject-specific random intercept that accounts for repeated REMS cycles within subject. For rodent data, analogous models were again fit separately by species and light/dark condition, with animal as the random intercept and no source term.

In both figures, the fitted coefficient of the predictor of interest (namely $\log(|\mathrm{REMpre}|)$ in Figure 2 and propensity P_{ij} in Figure 6) was interpreted as the primary measure of association. A positive coefficient indicates that larger predictor values are associated with larger expected values of the response after accounting for repeated observations within subject/animal. In the linear mixed-effects models, the random intercepts capture subject- or animal-specific baseline shifts, whereas the reported fixed-effect coefficient summarizes the population-level trend.

Robustness check: Gamma generalized linear mixed-effects models

As a robustness check against distributional assumptions, we also fit generalized linear mixed-effects models with Gamma response distribution and log link (Bolker et al., 2009). These models retain the same clustering structure as the linear mixed-effects models, but instead of modeling a log-transformed response, they model the conditional mean of the original positive response variable through a log link. Thus, for a response Y_{ij} (either $|\mathrm{IREM}|_{ij}$ in Figure 2 or $|\mathrm{REMpost}|_{ij}$ in Figure 6), the model takes the form

$$\log\big(\mathbb{E}[Y_{ij} \mid \text{predictors}, u_i]\big) = \eta_{ij},$$

where u_i is the subject- or animal-specific random intercept and η_{ij} is the corresponding linear predictor. In the human analyses, η_{ij} included the predictor of interest together with the categorical source effect and subject random intercept; in the rodent analyses, η_{ij} included the predictor of interest and animal random intercept only. These Gamma-log models are well suited for positive, right-skewed duration outcomes. Agreement between the linear mixed-effects and Gamma-log generalized linear mixed-effects results was taken as evidence that the conclusions were not sensitive to the particular modeling choice (see Tables S14 and S15).

Sensitivity analysis: subject-/animal-level aggregated Spearman correlation

As an additional sensitivity analysis, we aggregated the cycle-level data to the subject or animal level. Specifically, for each subject/animal we computed the median values of the two variables analyzed in the corresponding figure, and then assessed their association across subjects/animals using Spearman rank correlation (Spearman, 1961). For Figure 2, these variables were $|\mathrm{REMpre}|$ and $|\mathrm{IREM}|$. For Figure 6, they were REMS propensity and $|\mathrm{REMpost}|$. This approach is conservative because it removes within-subject replication entirely and reduces each subject or animal to a single summary point. Because Spearman correlation is rank-based, it is less sensitive to skewness and outliers than Pearson correlation.

S5.1 Robustness and sensitivity analyses for Figure 2

To assess whether the positive association shown in Fig. 2 between preceding REMS bout duration and the subsequent inter-REMS interval duration was robust to within-subject dependence and to the skewed distribution of inter-REMS interval duration, we performed three complementary analyses for each dataset: (i) a log-log linear mixed-effects model (LME), (ii) a Gamma generalized linear mixed-effects model with log link (GLME), and (iii) a subject-/animal-level aggregated Spearman correlation analysis. For the mixed-effects models, subject/animal was included as a random intercept, and, for the human dataset, data source was included as a fixed effect.

The LME analyses were fit in MATLAB using the function `fitlme`, and the Gamma-GLME analyses were fit using `fitglme`. In Tables S13 and S14, β denotes the estimated fixed-effect coefficient for $\log(|\mathrm{REMpre}|)$, that is, the change in the modeled outcome associated with a one-unit increase in $\log(|\mathrm{REMpre}|)$ after accounting for the other terms in the model. The reported 95% confidence intervals were obtained from the fitted models using MATLAB's `coefCI` function. The corresponding p-values were taken from the coefficient tables returned by `fitlme` and `fitglme`, respectively, and test the null hypothesis that the fixed-effect coefficient for $\log(|\mathrm{REMpre}|)$ is zero.

In Table S15, Spearman ρ denotes the rank-based correlation between subject-/animal-level aggregated values of preceding REMS bout duration and subsequent inter-REMS interval duration. Specifically, for each subject or animal, we computed the median values of $|\mathrm{REMpre}|$ and $|\mathrm{IREM}|$, and then assessed their association across subjects/animals using Spearman rank correlation. This analysis is conservative because it removes within-subject replication entirely and reduces each subject or animal to a single summary point. The corresponding p-value was obtained from MATLAB's `corr` function with `'Type','Spearman'` and tests the null hypothesis of no monotonic association.

Agreement across these three analyses was interpreted as evidence that the positive association in Fig. 2 was not an artifact of pseudo-replication or of the particular modeling assumptions used for the cycle-level data.

Table S13. Linear mixed-effects model results for the Fig. 2 analysis. The response was $\log(|\mathrm{IREM}|)$, with $\log(|\mathrm{REMpre}|)$ as the predictor of interest. Subject/animal was included as a random intercept; for the human dataset, source was included as an additional fixed effect.

Dataset	β (95% CI)		p value
Human	0.333	[0.281, 0.385]	2.92×10^{-35}
Mouse (Light)	0.381	[0.356, 0.407]	9.91×10^{-171}
Mouse (Dark)	0.434	[0.375, 0.494]	3.95×10^{-41}
Rat (Light)	0.334	[0.286, 0.381]	6.93×10^{-42}
Rat (Dark)	0.318	[0.269, 0.368]	6.14×10^{-35}

Table S14. Gamma generalized linear mixed-effects model results for the Fig. 2 analysis. The response was $|\mathrm{IREM}|$ (min), modeled with Gamma errors and a log link. Subject/animal was included as a random intercept; for the human dataset, source was included as an additional fixed effect.

Dataset	β (95% CI)		p value
Human	0.350	[0.297, 0.403]	1.19×10^{-37}
Mouse (Light)	0.347	[0.327, 0.367]	1.56×10^{-219}
Mouse (Dark)	0.373	[0.325, 0.421]	7.79×10^{-46}
Rat (Light)	0.289	[0.252, 0.326]	2.70×10^{-50}
Rat (Dark)	0.279	[0.240, 0.318]	2.78×10^{-42}

Table S15. Subject-/animal-level sensitivity analysis for the Fig. 2 relationship. For each subject/animal, observations were aggregated and the association between preceding REMS bout duration and subsequent inter-REMS interval duration was assessed using Spearman correlation across subjects/animals.

Dataset	n subjects/animals	Spearman ρ	p value
Human	515	0.458	5.61×10^{-28}
Mouse (Light)	179	0.690	1.26×10^{-26}
Mouse (Dark)	54	0.617	6.69×10^{-7}
Rat (Light)	44	0.564	6.58×10^{-5}
Rat (Dark)	37	0.550	4.22×10^{-4}

Across all datasets, the association between preceding REMS bout duration and subsequent inter-REMS interval duration remained positive and statistically significant in all three analyses. In the cycle-level mixed-effects analyses, the estimated coefficient for $\log(|\mathrm{REMpre}|)$ was positive in human, mouse light, rat light, mouse dark, and rat dark. The same qualitative result was obtained with the Gamma-log GLME, indicating that the observed association does not depend on assuming Gaussian residuals for a right-skewed outcome.

The subject-/animal-level aggregated Spearman analyses were also uniformly positive and significant across all datasets. This shows that the relationship is not solely driven by repeated cycles contributed by the same subject or animal, but is also preserved at the between-subject/animal level after aggregation.

Overall, these supplementary analyses strengthen the interpretation of Fig. 2 by showing that the positive relationship between preceding REMS bout duration and subsequent inter-REMS interval duration is robust across species, across light and dark phases in rodents, and across modeling assumptions.

S5.2 Robustness and sensitivity analyses for Figure 6

To evaluate whether the association shown in Fig. 6 was robust to within-subject dependence and to the skewed distribution of REMS bout duration, we performed three complementary analyses for each dataset: (i) a linear mixed-effects model (LME), (ii) a Gamma generalized linear mixed-effects model with log link (GLME), and (iii) a subject-/animal-level aggregated Spearman correlation analysis. For the mixed-effects models, subject/animal was included as a random intercept, and for the human dataset, recording source was included as a fixed effect. All analyses were restricted to REMS cycles lying in the increasing-propensity regime used in Fig. 6.

For Tables S16 and S17, β denotes the estimated effect of REMS propensity at REMS onset in the corresponding mixed-effects model, with the 95% confidence interval (CI) giving its uncertainty range and

the p value testing whether this effect differs from zero. In Table S18, Spearman ρ denotes the rank-based correlation computed from subject-/animal-level aggregated data between REMS propensity at REMS onset and the duration of the subsequent REMS bout, and the corresponding p value tests for the presence of a monotonic association.

Table S16. Linear mixed-effects model results for the Fig. 6 analysis. The response was $\log(|\mathrm{REMpost}|)$, with REMS propensity at REMS onset as the predictor of interest. Subject/animal was included as a random intercept; for the human dataset, source was included as an additional fixed effect.

Dataset	β (95% CI)		p value
Human	9.193	[4.217, 14.169]	3.06×10^{-4}
Mouse (Light)	3.531	[2.510, 4.551]	1.72×10^{-11}
Mouse (Dark)	0.314	[-0.922, 1.550]	0.618
Rat (Light)	3.536	[0.347, 6.726]	2.98×10^{-2}
Rat (Dark)	5.401	[1.028, 9.775]	0.0156

Table S17. Gamma generalized linear mixed-effects model results for the Fig. 6 analysis. The response was $|\mathrm{REMpost}|$ (seconds), modeled with Gamma errors and a log link. Subject/animal was included as a random intercept; for the human dataset, source was included as an additional fixed effect.

Dataset	β (95% CI)		p value
Human	7.595	[3.216, 11.974]	6.97×10^{-4}
Mouse (Light)	2.880	[1.947, 3.814]	1.82×10^{-9}
Mouse (Dark)	-0.320	[-1.425, 0.784]	0.569
Rat (Light)	4.196	[1.323, 7.069]	4.30×10^{-3}
Rat (Dark)	5.207	[1.323, 9.090]	8.73×10^{-3}

Table S18. Subject-/animal-level sensitivity analysis for the Fig. 6 relationship. For each subject/animal, observations were aggregated and the association between REMS propensity and subsequent REMS bout duration was assessed using Spearman correlation across subjects/animals.

Dataset	n subjects/animals	Spearman ρ	p value
Human	515	0.185	2.85×10^{-4}
Mouse (Light)	179	0.357	1.03×10^{-6}
Mouse (Dark)	54	-0.086	0.537
Rat (Light)	44	-0.099	5.21×10^{-1}
Rat (Dark)	37	0.046	0.786

Overall, the mixed-effects analyses support a positive association between REMS propensity at REMS onset and the duration of the subsequent REMS bout in all three datasets shown in Fig. 6, namely human, mouse (light phase), and rat (light phase) data sets. In the LME analysis, the estimated propensity coefficient was positive and statistically significant. The same qualitative conclusion was obtained with the Gamma GLME, indicating that the observed association is not an artifact of applying a Gaussian model to a right-skewed outcome.

All three analyses gave consistent results for the human data. In addition to the positive and statistically significant effect of propensity in the mixed-effects models, the subject-level Spearman analysis also

remained positive and significant. This indicates that the relationship is present both at the cycle level, after accounting for repeated observations within subjects, and at the coarser subject-aggregated level.

The analyses for the mouse (light phase) data were likewise highly consistent and, among the three datasets, arguably the strongest. The propensity effect remained positive and highly significant in both the LME and Gamma GLME analyses, and the subject-level Spearman correlation was also positive and significant. Thus, the mouse light-phase result appears robust to model choice and to aggregation across repeated cycles.

Analysis results for the rat (light phase) data were weaker but still supportive at the cycle level. In both mixed-effects models, the estimated propensity effect was positive and statistically significant. However, the subject-level aggregated Spearman correlation was not significant. This suggests that, although the cycle-level association is detectable after accounting for repeated measurements, the between-animal trend is less stable in the rat light-phase data than in the human and mouse light-phase datasets.

Analogous analyses were also performed for the rodent dark-phase datasets. For the mouse dark-phase data, the association between REMS propensity at onset and subsequent REMS bout duration was not supported by any of the three analyses, indicating no clear evidence for a positive relationship in this condition. For the rat dark-phase data, the cycle-level mixed-effects analyses supported a positive association, but this pattern was not retained in the subject-level aggregated sensitivity analysis. Overall, the dark-phase results were less consistent than those obtained for the light-phase datasets shown in Fig. 6, with mouse dark-phase data showing a null pattern and rat dark-phase data showing only partial support for a positive association.

Taken together, these supplementary analyses strengthen the interpretation of Fig. 6. They show that the positive association between REMS propensity and subsequent REMS bout duration is robust in human and mouse data, and is also supported at the cycle level in rat data, while appearing less consistent at the aggregated between-animal level in the rat dataset.

REFERENCES

Bates, D., Mächler, M., Bolker, B., and Walker, S. (2015). Fitting linear mixed-effects models using lme4. *Journal of statistical software* 67, 1–48

Bolker, B. M., Brooks, M. E., Clark, C. J., Geange, S. W., Poulsen, J. R., Stevens, M. H. H., et al. (2009). Generalized linear mixed models: a practical guide for ecology and evolution. *Trends in ecology & evolution* 24, 127–135

Pinheiro, J. C. and Bates, D. M. (2000). *Mixed-effects models in S and S-PLUS* (Springer)

Spearman, C. (1961). The proof and measurement of association between two things.

Stute, W., Manteiga, W. G., and Quindimil, M. P. (1993). Bootstrap based goodness-of-fit-tests. *Metrika* 40, 243–256
